# Development of crystal optics for X-ray multi-projection imaging for synchrotron and XFEL sources

**DOI:** 10.1107/S1600577524008488

**Published:** 2024-10-21

**Authors:** Valerio Bellucci, Sarlota Birnsteinova, Tokushi Sato, Romain Letrun, Jayanath C. P. Koliyadu, Chan Kim, Gabriele Giovanetti, Carsten Deiter, Liubov Samoylova, Ilia Petrov, Luis Lopez Morillo, Rita Graceffa, Luigi Adriano, Helge Huelsen, Heiko Kollmann, Thu Nhi Tran Calliste, Dusan Korytar, Zdenko Zaprazny, Andrea Mazzolari, Marco Romagnoni, Eleni Myrto Asimakopoulou, Zisheng Yao, Yuhe Zhang, Jozef Ulicny, Alke Meents, Henry N. Chapman, Richard Bean, Adrian Mancuso, Pablo Villanueva-Perez, Patrik Vagovic

**Affiliations:** a European XFEL GmbH Schenefeld Germany; b SmarAct GmbH, Oldenburg, Germany; c ESRF – European Synchrotron Radiation Facility, Grenoble, France; d Integra TDS Ltd, Piestany, Slovakia; e Institute of Electrical Engineering, Bratislava, Slovakia; f University of Ferrara, Ferrara, Italy; g INFN – Istituto Nazionale di Fisica Nucleare Ferrara Italy; h Synchrotron Radiation Research and NanoLund, Lund University, Sweden; i University of P. J. Safarik, Kosice, Slovakia; j Center for Free-Electron Laser Science (CFEL), DESY, Hamburg, Germany; k Diamond Light Source Harwell Science and Innovation Campus DidcotOX11 0DE United Kingdom; l Department of Chemistry and Physics, La Trobe Institute for Molecular Science, La Trobe University, Melbourne, Victoria3086, Australia; m University of Hamburg, Hamburg, Germany; Paul Scherrer Institut, Switzerland

**Keywords:** X-ray imaging, multi-projection, crystal splitter, XFEL, fast 3D imaging

## Abstract

X-ray multi-projection imaging (XMPI) is an emerging experimental technique for the acquisition of rotation-free, time-resolved, volumetric information on stochastic processes. The technique is developed for high-brilliance light-source facilities, aiming to address known limitations of state-of-the-art imaging methods in the acquisition of 4D sample information, linked to their need for sample rotation. XMPI relies on a beam-splitting scheme, that illuminates a sample from multiple, angularly spaced viewpoints, and employs fast, indirect, X-ray imaging detectors for the collection of the data. This approach enables studies of previously inaccessible phenomena of industrial and societal relevance such as fractures in solids, propagation of shock waves, laser-based 3D printing, or even fast processes in the biological domain. In this work, we discuss in detail the beam-splitting scheme of XMPI. More specifically, we explore the relevant properties of X-ray splitter optics for their use in XMPI schemes, both at synchrotron insertion devices and XFEL facilities. Furthermore, we describe two distinct XMPI schemes, designed to faciliate large samples and complex sample environments. Finally, we present experimental proof of the feasibility of MHz-rate XMPI at the European XFEL. This detailed overview aims to state the challenges and the potential of XMPI and act as a stepping stone for future development of the technique.

## Abstract 2

X-ray multi-projection imaging (XMPI) is an emerging technology that enables the acquisition of millions of 3D images per second, useful for observing rapid, stochastic phenomena previously inaccessible to conventional tomography. This study explores XMPI schemes and optics compatible with synchrotron and XFEL beams, and it experiments with MHz-rate XMPI at the European XFEL.

## 1. Introduction

Numerous rapid and stochastic phenomena with significant industrial and societal implications take place in materials opaque to visible light. These phenomena include the propagation of shock waves (Prasad *et al.*, 2016 ▸; Grady, 1998 ▸), fractures in stressed solids (Kumar *et al.*, 2016 ▸; Xu *et al.*, 2020 ▸), laser 3D printing (Chen *et al.*, 2020 ▸; Hocine *et al.*, 2020 ▸), surface peening (Soyama & Korsunsky, 2022 ▸; John *et al.*, 2021 ▸; Soyama & Iga, 2023 ▸) and fast biological processes (Hansen *et al.*, 2021 ▸; Truong *et al.*, 2020 ▸). Investigating and understanding these complex events is complicated by the absence of a suitable 3D imaging technique with microsecond time resolution.

**Figure 1:**
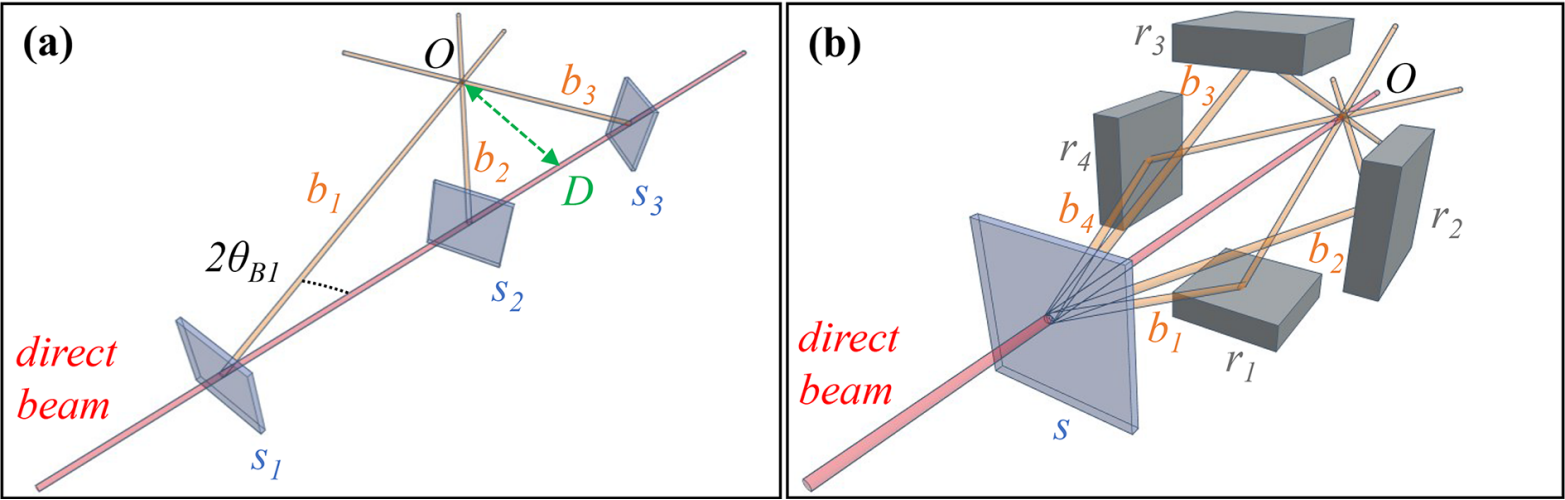
Descriptive sketches of the In-Line (*a*) and In-Parallel (*b*) multi-projection schemes. (*a*) Multiple crystal beam-splitters (*s*_1_, *s*_2_, *s*_3_) are placed on the direct beam path. Each splitter diffracts a single beamlet (*b*_1_, *b*_2_, *b*_3_) out of the direct beam at an angle equal to twice the Bragg angle (2θ_B1_ for the first splitter). The type, position and orientation of the beam-splitters are chosen such that the beamlets converge to a point where the sample object is placed *O* at a distance *D* from the direct beam. (*b*) A single beam-splitter *s* is oriented in the direct beam to excite multiple Bragg diffractions producing several beamlets (4 in the example *b*_1_, *b*_2_, *b*_3_, *b*_4_). The beamlets are diffracted by recombiner crystals (*r*_1_, *r*_2_, *r*_3_, *r*_4_) towards a common point *O* on the direct beam path where the sample object is placed.

One promising technique for probing such systems is fast 3D X-ray microscopy. The current state of the art in fast single-projection radiography is primarily limited by the X-ray source’s flux and the capabilities of the detector. Recent developments have enabled the attainment of MHz frame rate radiography at synchrotron facilities (Fezzaa & Wang, 2008 ▸; Olbinado *et al.*, 2017 ▸) and X-ray free-electron laser (XFEL) sources (Vagovič *et al.*, 2019 ▸). However, when it comes to tomography techniques, the time resolution is primarily constrained by technical considerations such as centrifugal forces, with current rates reaching up to 1 kHz in synchrotron experiments (García-Moreno *et al.*, 2021 ▸). Centrifugal forces pose a significant technological challenge for the instrumentation and a fundamental challenge for the sample since the shear forces can disrupt the sensitive dynamics under investigation. Rotation-free kHz- and MHz-rate 3D X-ray imaging may be attained by X-ray multi-projection imaging (XMPI) schemes. These schemes leverage Bragg crystal optics to split the incoming X-ray beam into multiple beamlets, allowing the sample to be examined simultaneously from different angles. Subsequently, a 3D representation of the sample is reconstructed using these multiple views, as demonstrated, for instance, by Zhang *et al.* (2023 ▸). With the centrifugal forces excluded from the system, the maximum acquisition rate would be determined by the luminosity of the setup. Therefore, it may be possible to achieve MHz-rate 3D X-ray imaging at XFEL sources and kHz-rate at synchrotrons. In this context, the European XFEL is a prime candidate for achieving MHz-rate 3D X-ray imaging because of the high flux per pulse and MHz repetition rate of the source. There have been developments towards 3D kHz imaging at synchrotrons based on XMPI systems (Villanueva-Perez *et al.*, 2018 ▸; Voegeli *et al.*, 2020 ▸; Bellucci *et al.*, 2023 ▸). The wavefront of a large white beam can be divided into dozens of small beamlets (Voegeli *et al.*, 2020 ▸). This method cannot be used with X-ray beams of small size when imaging a sample of comparable size. Therefore, a mm-size XFEL beam would require an amplitude division system to image a mm-size sample. The amplitude of a small beam can be divided into multiple, virtually identical monochromatic beamlets by using a single beam-splitter positioned to create multiple beamlets by Bragg diffraction (Villanueva-Perez *et al.*, 2018 ▸). In this case, the coincidence point of the system is in the splitter itself so the sample must be placed as close as possible to the splitter, which limits the size of the sample environment. Here we describe two possible schemes (Vagovic *et al.*, 2023 ▸; Villanueva-Perez *et al.*, 2023 ▸), designed to overcome those drawbacks and permit larger samples and more complex sample environments, focusing on the crystal optics and related instrumentation. These two schemes are referred to here as In-Line [Fig. 1 ▸(*a*)] and In-Parallel [Fig. 1 ▸(*b*)] multi-projection geometries. Both schemes rely on amplitude splitters, *i.e.* single elements that divide the amplitude of a beam. The In-Parallel scheme is based on a multi-wave Laue crystal (Villanueva-Perez *et al.*, 2018 ▸) while the In-Line scheme is based on a novel in-line configuration of crystal splitters (Vagovic *et al.*, 2023 ▸). Both configurations have advantages as the In-Parallel configuration works efficiently with a monochromatic beam (as an XFEL seeded beam with 1 eV bandwidth) while the In-Line configuration works better with broader band sources (20 eV bandwidth) and it is tunable in photon energy.

The In-Parallel scheme was employed at the TOMCAT beamline of the Swiss Light Source of the Paul Scherrer Institut (PSI) and the experimental demonstration is discussed in Section 6.1. The In-Line scheme has been commissioned at the Single Particles, Clusters and Biomolecules and Serial Femtosecond Crystallography (SPB/SFX) instrument of the European XFEL (EuXFEL), as discussed in Section 6.2 (see also Villanueva-Perez *et al.*, 2023 ▸), and the ID19 beamline of the European Synchrotron Radiation Facility (ESRF) (Asimakopoulou *et al.*, 2024 ▸).

The setups have been supported by simulations that resulted in the requirements for the X-ray optics (Section 3), their development (Section 4) and their subsequent characterization (Appendix *D*[App appd]). The performance of the presented schemes requires accurate and stable positioning of the optics. This led to the development and testing of high-precision mechatronics, which are discussed in Appendix *C*[App appc].

## 2. In-Line multi-projection geometry

The In-Line multi-projection scheme geometry is defined by multiple crystal beam-splitters placed sequentially into the path of the incident beam. The parameters, location and orientation of each crystal splitter are chosen such that a part of the beam is selected and transmitted to a single interaction point where the sample environment is placed. Assuming a right-handed *XYZ* coordinate system, with *Z* being along the beam direction (positive direction downstream), *Y* vertical with respect to the optical table and *X* perpendicular to the beam direction, the position *P* of each splitter along *Z* is easily calculated,
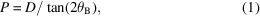
where *D* is the minimum distance along *X* between the sample and the direct beam and θ_B_ is the Bragg angle of the splitter. The (0, 0, 0) point is assumed to be along the beam direction, at the point of orthogonality with the sample position. The diffraction angles of the crystals are set in the horizontal plane.

### 2.1. Crystal splitter design simulations

The purpose of a crystal splitter is to divert a large portion of the direct beam into the diffracted branches (beamlets) while absorbing a small fraction of the direct beam so that the beam-splitter downstream intercepts an intense beam. The design of a splitter takes into consideration the following parameters: (1) transmission, (2) size of the diffracted beam (field of view), (3) stiffness of the splitter, (4) diffracted intensity and (5) manufacturing limitations. Here we investigate splitters fabricated as diamond, silicon and germanium monocrystals since (*a*) it is relatively easy to source high-perfection single crystals of these elements, and (*b*) these cover a wide range of electron densities, absorption and diffraction intensity.

In this study, we take the photon energies of 8, 10 and 15 keV as examples because (i) this range of energy allows studies in mm-size samples with absorption levels from plastic to aluminium; (ii) the integrated diffraction efficiency of the splitters is about halved from 8 to 15 keV; (iii) the angles between the beamlets from the same diffraction planes are also halved from 8 to 15 keV, which may decrease the quality of 3D reconstructions (Zhang *et al.*, 2023 ▸). One might increase the angles between the beamlets (iii) by using diffraction planes of higher order but at the cost of worsening the integrated diffraction efficiency (ii). Diffraction planes are indicated with the material symbol followed by the Miller index of the plane, *e.g.* C111 is the diamond diffraction plane (111).

#### 2.1.1. Transmission

**Figure 2:**
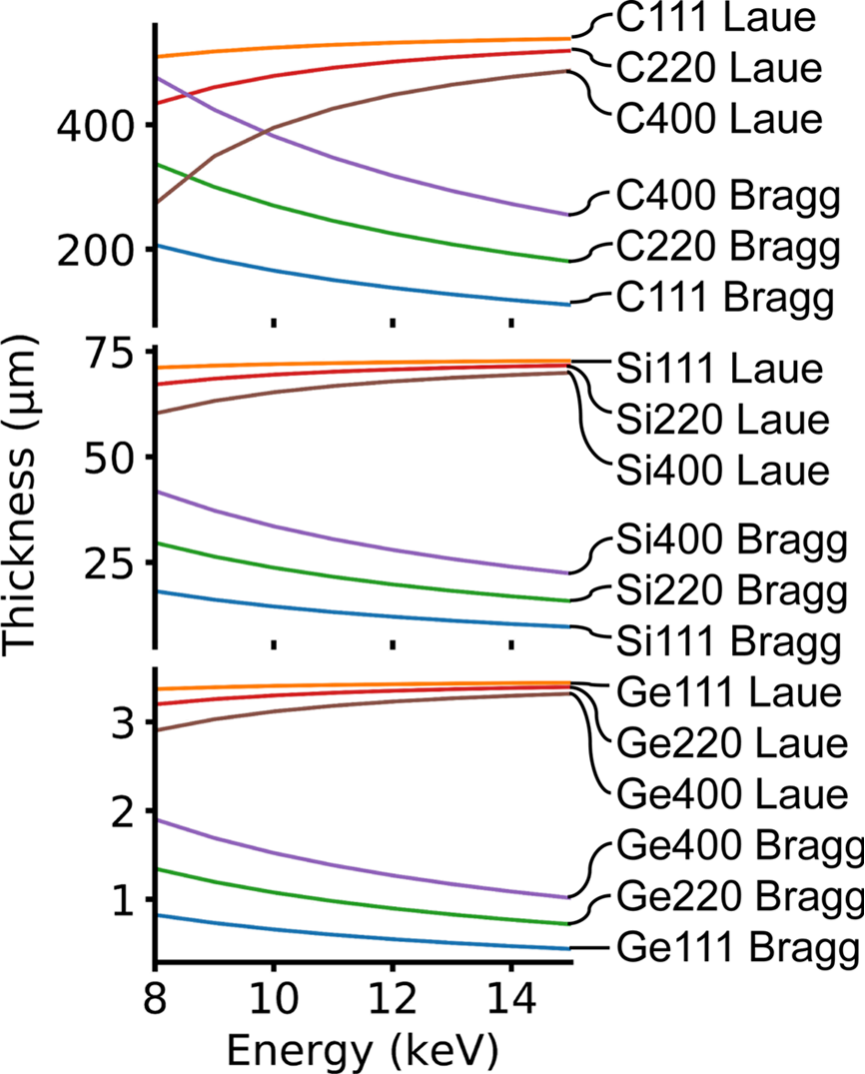
Beam-splitter thickness versus energy for a resulting 90% transmitted direct beam, when traversing a beam-splitter in symmetric Laue or Bragg diffraction geometry, for different selected materials (diamond C, silicon Si, germanium Ge) and diffraction planes (111), (220), (400) in order of diffraction intensity. The selected range of photon energies 8–15 keV is where the In-Line geometry can operate best.

The transmission of each beam-splitter should allow sufficient incident intensity at downstream splitters. A threshold of a minimum 90% transmission is chosen here. The transmission *I*_T_ of the direct beam is calculated as
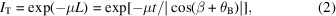
with μ the linear absorption coefficient of the material, *L* the length of the crystal traversed by the direct beam, *t* the thickness of the splitter, θ_B_ the Bragg angle, β is the angle between the normal to the crystal surface and the trace of the lattice planes (Appendix *A*[App appa]). β = 0 for symmetric Laue geometry and β = −π/2 for symmetric Bragg geometry. In the case of symmetric Laue or Bragg geometry, the traversed length *L* can be reduced to
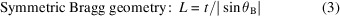

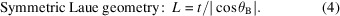
In the following calculations, we always assume symmetric Bragg and Laue geometry because asymmetric diffraction produces a magnification of the diffracted beam. This effect can be used for adjusting the size of the diffracted beam to the field of view of the detector system, as well as for adjusting the passband of the diffraction plane. However, this treatment is too specific to the detector system used in the particular setup; therefore it will not be treated here. This magnification effect is treated in Appendix *B*[App appb] and used in the In-Parallel setup since multiple beam-splitting inherently requires asymmetric diffraction planes. A plot of splitter thickness *t* versus energy at a 90% transmission condition is represented in Fig. 2 ▸ for selected materials and diffraction planes.

#### 2.1.2. Field of view

The size of the diffracted beams (field of view) should match the maximum sample size that the specific beamline can accept. In this instance, the optimization is carried out for the EuXFEL’s SPB/SFX instrument, which has a maximum beam size of 3 mm × 3 mm. In the horizontal scattering geometry, the vertical footprint of the beam on the crystal is equal to the beam height, while the horizontal footprint is a function of the Bragg angle,
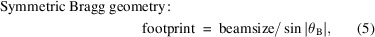

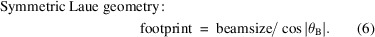
The maximum footprints occur for Bragg (111) diffraction at the highest energy (15 keV).

#### 2.1.3. Stiffness

A stiff splitter reduces vibrations that may affect imaging. For a slab of uniform material, the stiffness is proportional to the cube of the thickness while the momenta are proportional to its size (Landau & Lifshitz, 1986 ▸), so the stiffness is maximized by reducing the area while increasing the thickness. Therefore, the splitter thickness should be maximized and its area minimized while keeping transmission (1) above 90%, a large field of view (2), a high diffraction efficiency (4).

#### 2.1.4. Diffracted intensity

**Figure 3:**
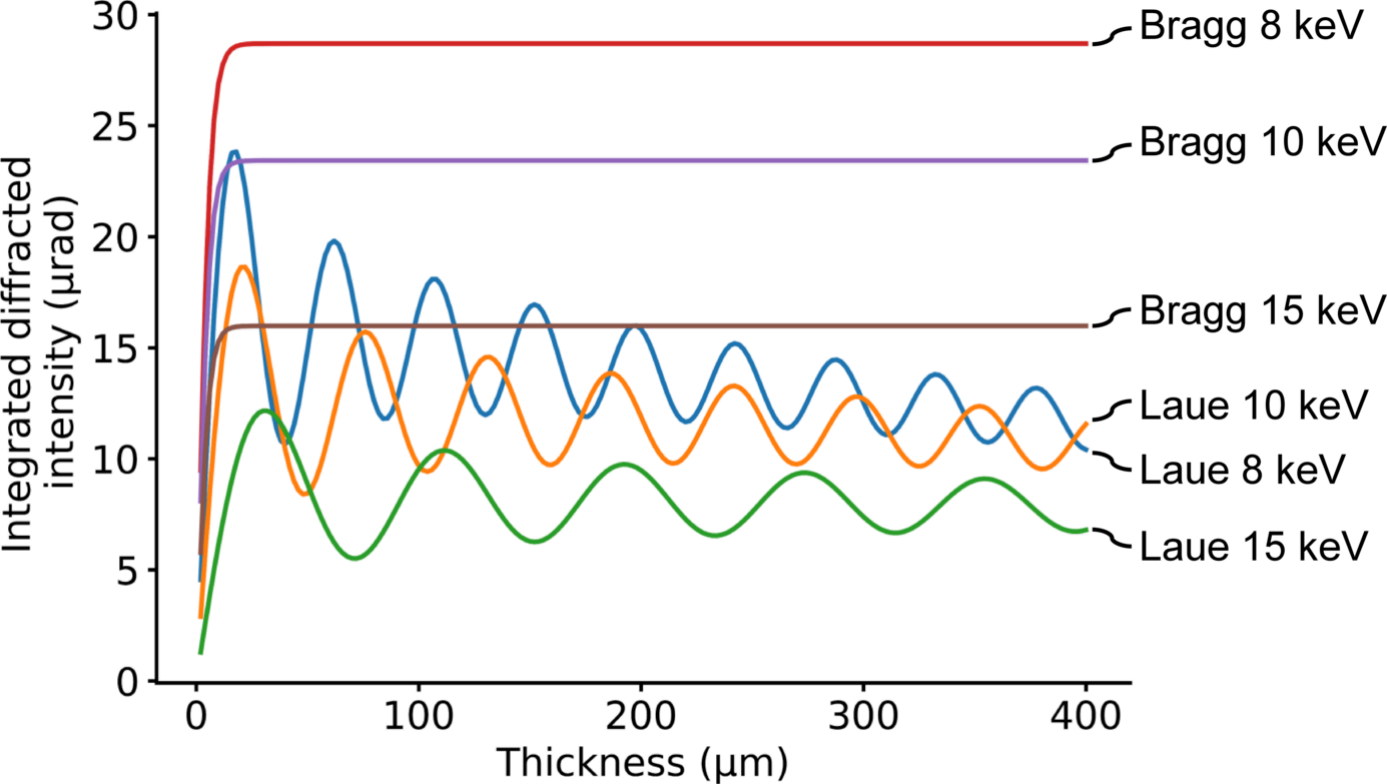
Integrated diffracted intensity versus beam-splitter thickness for a diamond splitter diffracting via its (111) symmetric Laue or Bragg lattice planes, for different selected photon energies 8, 10 and 15 keV. Laue geometry presents symmetry between the diffracted and transmitted beams, which results in oscillations in the diffracted intensity.

A splitter should diffract a large portion of the beam; therefore, we optimize the total intensity diffracted by the splitter (integrated diffracted intensity 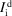) versus the thickness of the splitter as per the dynamical theory of X-ray diffraction (Authier, 2001 ▸). Splitter diffraction in Laue or Bragg geometry follows different functions (Appendix *A*[App appa]), so the two cases must be studied separately (Fig. 3 ▸). In both cases, we consider symmetric diffraction geometries.

The 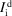 function versus the thickness of the Laue splitters follows an oscillatory pattern (Fig. 3 ▸), with the absolute maximum always reached on the first peak, *i.e.* the peak with lowest thickness. However, this low thickness may conflict with the technical realization of the splitter (5) and with optimization of its stiffness (3). Moreover, the designed splitter must work for a range of photon energies, but the period of the oscillation changes broadly with the energy; thus, after the first peak, it is not possible to detect a peak common for the different energies. Therefore, after the first peak, the best option is to increase the thickness to up to where the oscillations stabilize around an average due to the statistical nature of the *Pendellösung* oscillations.

In the symmetric Bragg case, 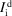 converges rapidly to an average where oscillations are negligible. On average, the integrated diffracted intensity in Bragg geometry is about 50% higher than in Laue geometry.

#### 2.1.5. Manufacturing limitations

The technical difficulty of realizing crystal splitters increases with thickness < 200 µm. Silicon splitters of thickness ∼10 µm are commercially available, but such a low thickness allows for warping issues under the heating provoked by an intense X-ray beam (Asimakopoulou *et al.*, 2024 ▸). Indeed, diamond crystals are preferable for high-heat-load applications (Van Vaerenbergh *et al.*, 2010 ▸; Tasca *et al.*, 2022 ▸). The technology for producing dislocation-free diamonds is currently limited to a 3 mm × 3 mm clear optic area, *i.e.* an area free of any dis­location or inclusion (Samoylova *et al.*, 2019 ▸; Kaganer *et al.*, 2021 ▸). Therefore, this is the upper area limit for diamond splitters. For silicon and germanium, this technological limit does not exist, so it is possible to accommodate the entire footprint of the beam. The quality of germanium single-crystal ingots is good enough for coherent applications, as demonstrated by Vagovič *et al.* (2014 ▸) and Spiecker *et al.* (2023 ▸).

### 2.2. Diamond, silicon and germanium splitters

Applying the simulations for the different materials, and lattice planes, and balancing the points (1) to (5), we can obtain the splitter dimensions (Table 1 ▸).

#### 2.2.1. Diamond splitters

The best dimensions simulated for Laue diamond splitters are a thickness of around 100 µm (Table 1 ▸) according to (1), (3), (4), (5) and an optic area of 3 mm × 3 mm according to (2) and (5). For Bragg splitters, the optimal thickness varies more, ranging from 25 µm for C111 to 80 µm for C440, as it increases with the Miller index.

#### 2.2.2. Silicon splitters

For Laue silicon splitters, the best dimensions simulated are a thickness of around 10 µm and horizontal size of 5 mm, while for Bragg splitters the thickness ranges from 3 to 10 µm with changing Miller index and horizontal size up to 23 mm. The vertical dimension stays fixed at 3 mm to maximize the stiffness (3). The low thickness required is a technological challenge and the first tests of a thin silicon beam-splitter are shown in the experimental part of the paper (Section 6).

#### 2.2.3. Germanium splitters

For a Laue germanium splitter, the best simulated thickness is about 4 µm, while for a Bragg splitter, it is below 1 µm. Together with the brittle nature of germanium, the low thickness makes this splitter technologically not feasible. Therefore, germanium can be used just as a thick crystal positioned last in the In-Line setup, so its high absorption does not affect other splitters. The size of the optic area is not limited by technology, so it can be up to 19 mm horizontally and 3 mm vertically (2).

## 3. In-Parallel multi-projection geometry

The In-Parallel multi-projection scheme geometry is defined by a single-crystal beam-splitter placed on the direct beam path and an ensemble of beam recombiners placed in a conical symmetry around the direct beam path. As for the previously described In-Line geometry, the parameters, location and orientation of each crystal are chosen such that a part of the beam is diffracted and recombined to a single interaction point where the sample environment is placed.

### 3.1. Beam-splitter simulations

The purpose of the beam-splitter in the In-Parallel geometry is to produce diffracted beamlets in a conical geometry (Villanueva-Perez *et al.*, 2018 ▸). To this end, the beam-splitter was selected between families of lattice planes having cylindrical symmetry (Table 2 ▸), which can divide an X-ray beam into identical beamlets by multiple Bragg diffraction. Selecting one of these families means fixing the diffraction energy since the Bragg angle is the inclination angle of the plane’s family. For our setup, we selected a splitter with (100) main face and Laue diffraction planes of the 113 family, having a 17.55° asymmetry angle. This geometry is valid both for diamond and silicon splitters since these two elements have the same crystal structure (diamond cubic). Nevertheless, diamond and silicon have different lattice parameters, which results in different working energy, 12.4 keV for silicon and 19.1 keV for diamond.

This particular splitter was selected between the combinations available in Table 2 ▸ because (i) the photon energy is compatible with the maximum flux of EuXFEL (8–20 keV), (ii) with a 19.1 keV X-ray beam, it is possible to traverse mm-size aluminium samples, where aluminium alloys are important industrial materials for crack propagation studies, (iii) the 35.1° 2θ diffraction angle is relatively large, allowing for a compact and portable system, (iv) the 113 family allows for splitting into eight beams, enabling the expansion of the system to up to eight beamlets, (v) both diamond and silicon have low X-ray absorption, and (vi) it is technologically possible to realize perfect diamond or silicon crystals of at least mm size. Between silicon and diamond, the latter was selected as the best candidate for XFELs due to the lower absorption and larger thermal conductivity, which enable it to better withstand the intense XFEL beam. Silicon is better suited for synchrotrons since it provides a larger diffracted intensity in an environment where the thermal load is less critical. For lower photon energies, a splitter with (110) main face and diffraction planes of the silicon 220 family at 6.5 keV or diamond 220 family at 9.8 keV is preferable because the (220) diffraction has a larger Darwin width [equation (13)] than the (113) diffraction, therefore diffracting a higher flux into the beamlets. It is important to point out that the choice of the optimal splitter parameters and working energy changes between the In-Line and In-Parallel geometry because of the different requirements of these two geometries.

### 3.2. Recombiner simulations

Selecting the recombiners [Fig. 1 ▸(*b*)] also involved iterating through materials and diffraction planes, this time focusing on three points:

#### 3.2.1. Angle of view between two opposing beamlets

The angle of view θ_V_ between two opposing beamlets should be as close to 90° as possible to ease 3D reconstruction (Zhang *et al.*, 2023 ▸). It can be easily calculated by ray tracing from the Bragg angles of the beam-splitter θ_Bs_ and recombiners θ_Br_ by
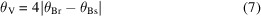
as shown in Table 3 ▸ for different materials and diffraction planes.

#### 3.2.2. Diffraction efficiency

**Figure 4:**
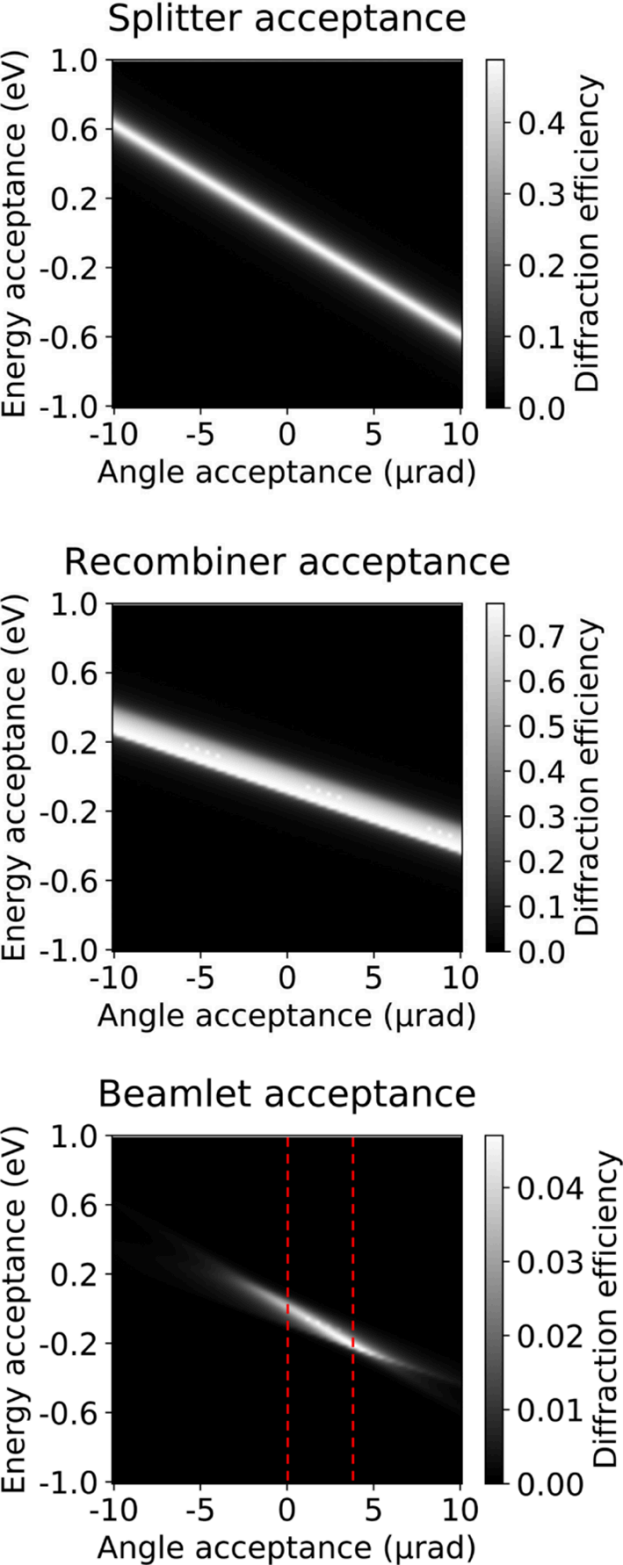
Simulation of the In-Parallel multi-projection setup acceptance using DuMond diagrams showing diffraction efficiency versus angle and photon energy. The splitter and recombiner acceptances are represented by bands with different widths and inclinations. In this example, the splitter is a diamond 113 Laue with asymmetry 17.55°, while the recombiner is a germanium (660) Bragg with asymmetry angle 10° and multiplicity 8. The beamlet acceptance is obtained by multiplying the DuMond diagrams of the splitter and recombiner and dividing by the multiplicity of the plane family. The direct beam is visible in the last graphic as dashed red lines, in this example with photon energy 19.1 keV, chromaticity 20 eV and angular divergence 4 µrad to simulate the EuXFEL SPB/SFX beam. This photon energy and the splitter parameters were selected for the reasons listed in Sections 3.1 and 3.2.

Diffraction efficiency is calculated from the dynamical theory of X-ray diffraction [equation (12)] (Authier, 2001 ▸). The acceptance and diffraction efficiency of a crystal with respect to a range of photon energies and a range of incidence angles can be expressed by a DuMond diagram (DuMond, 1937 ▸; Davis, 1990 ▸; Authier, 2001 ▸). Fig. 4 ▸ illustrates the DuMond diagrams for the splitter, the recombiner and the combination of these two elements. The integrated diffraction efficiency for each beamlet is obtained by integrating the beamlet acceptance over the chromaticity and divergence of the beam, resulting in 0.78 × 10^−4^ for the example in Fig. 4 ▸. The recombiner must be designed in such a way that its passband accepts a large fraction of the beam diffracted by the splitter. This can be achieved by a wide angular acceptance θ_A_,
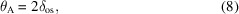
where δ_os_ is the Darwin width [equation (13)]. The acceptance usually increases for heavier materials as it depends on the electron density. For the recombiners, transmission is not a design parameter and concerns about thermal load are greatly relaxed since a recombiner intercepts just a beamlet, which contains less than 1% of the direct beam flux. This holds true in general for the second crystal in a monochromator (Carpentier *et al.*, 2001 ▸; Macrander *et al.*, 1992 ▸). Therefore, we can choose heavier materials, *i.e.* silicon or germanium versus diamond.

Asymmetry can be used for enlarging the acceptance of the recombiners [equation (13)] (Authier, 2001 ▸) while enlarging the physical size of the diffracted beamlet over the diffraction direction by a magnification factor *M* [equation (15)]. Enlarging the beamlet’s physical size can be beneficial since the beamlet was already shrunk due to the asymmetry of the splitter. Indeed, the total magnification of the beamlet is obtained by multiplying the magnifications produced by the splitter and the recombiner. Therefore, we can select a recombiner’s asymmetry that increases the acceptance while making the shape of the beamlet more symmetric, or similar to the shape of the field of view of the camera. For our specific setup, the target camera is the MHz camera Shimadzu HPV-X2. Details of the treatment for this case can be found in Appendix *B*[App appb], resulting in a 10° asymmetry angle for the germanium recombiners.

#### 3.2.3. Ease of alignment and stability of the system after alignment

The ease of alignment and the stability of the system following alignment is critical since the beam is diffracted by the splitter and is narrow in chromaticity and divergence, on the order of 10^−4^. Therefore, a small misalignment can degrade the diffraction condition. To simplify the alignment, germanium is the most suitable material for the recombiners, having twice the acceptance of silicon and multiple times that of diamond. A grazing asymmetry of 10° further increases the acceptance.

#### 3.2.4. Selection of the recombiners

All considerations presented above lead to the selection of germanium recombiners, main face (110) with 10° asymmetry. The germanium 110 family can provide a degree of flexibility at several photon energies (Table 3 ▸) enabling a range of angles of view including those close to 90°.

## 4. Realization of the crystals

**Figure 5:**
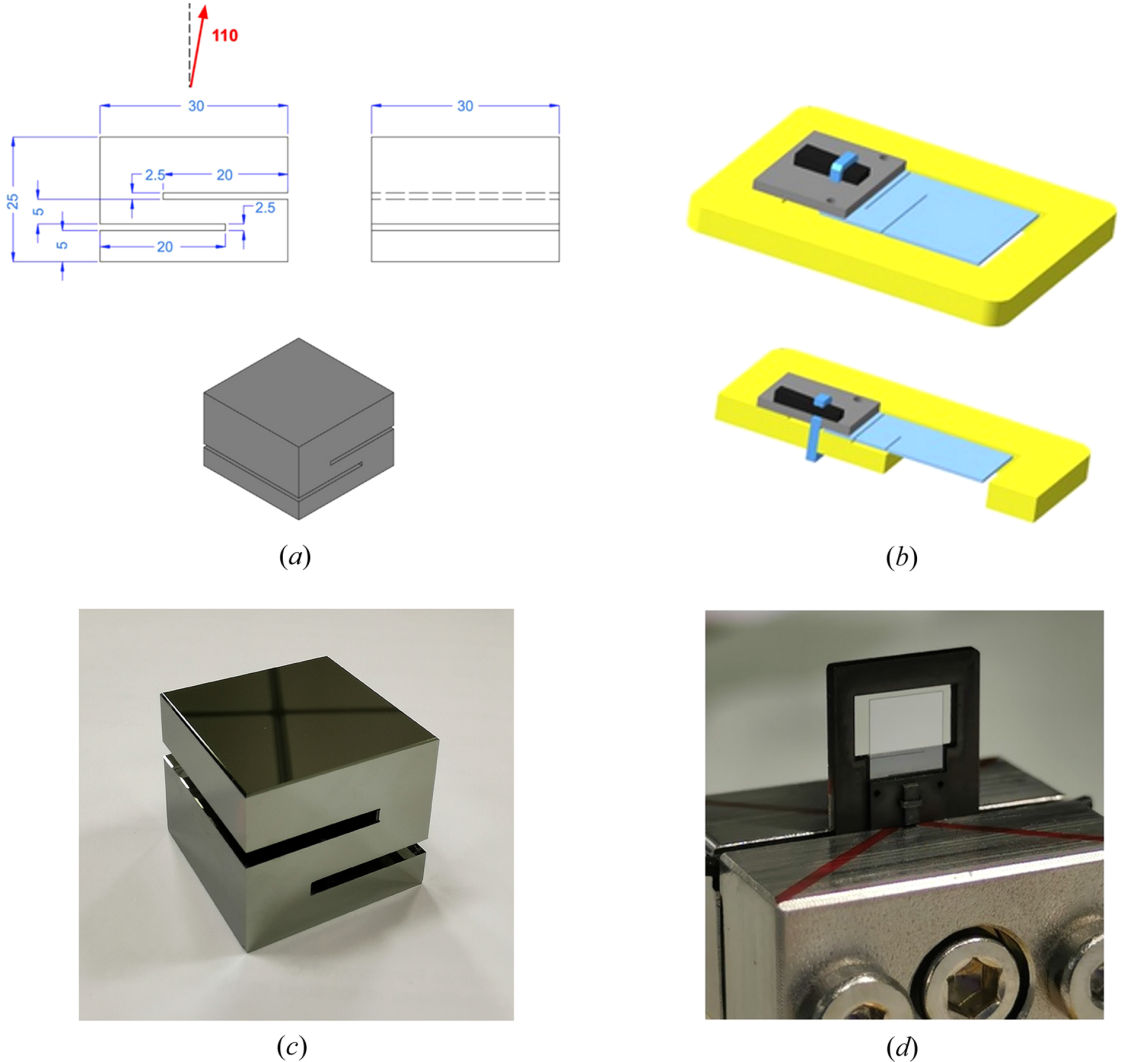
Crystals used in the multi-projection setup. (*a*) Drawing and (*c*) picture of a recombiner. The 2.5 mm large stress relief cuts are visible, giving an S-shape to the profile of the crystal. (*b*) Drawing of a diamond beam-splitter, light blue being the actual beam-splitter, yellow the polycrystalline frame and gray graphite used for fixing the two together. Two stress relief cuts are visible on the base of the beam-splitter near the clamping point with the graphite. (*d*) Picture of a mounted beam-splitter. Both for the recombiners and the splitters, the stress relief cuts prevent the stress from clamping to propagate to the optic area of the crystal.

The specifications of the crystals were a trade-off between design requirements and technological feasibility. The current technology for producing monocrystalline diamonds (high-pressure high-temperature diamonds) allows for reliable production of slabs free of dislocations with an area of 3 mm × 3 mm or smaller (Samoylova *et al.*, 2019 ▸; Kaganer *et al.*, 2021 ▸), so this is the maximum size of the optic area. The remaining non-perfect part of the slab is used for the strain relief cuts and holding section. Diamond crystals are protected by a frame made of polycrystalline diamond to ease thermal dispersion. The splitter is fixed to the frame by the bottom part of the strain relief section to avoid any strain in the optic part (Fig. 5 ▸).

**Figure 6:**
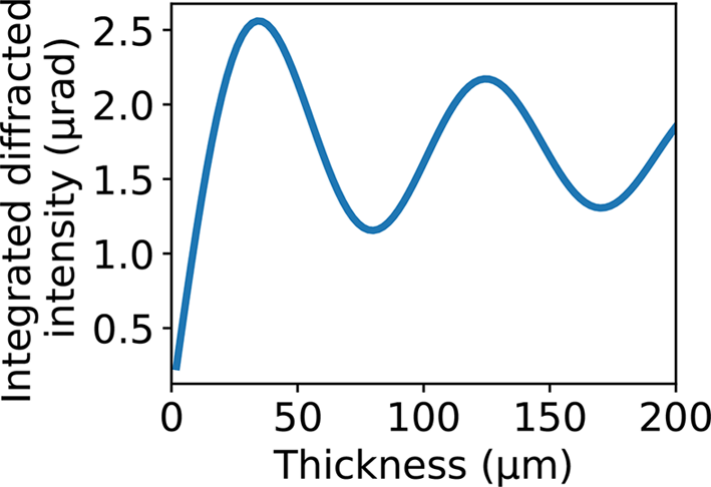
Integrated intensity versus thickness for the diamond splitter of the In-Parallel geometry, (113) diamond diffraction plane with 17.5° asymmetry at 19.1 keV.

The In-Parallel splitter was realized with a 130 µm thickness. This value was chosen since it is one of the thicknesses for which the integrated diffracted intensity shows a peak value for the selected 113 diffraction plane family, while the absorption is low, as shown in Fig. 6 ▸. The thickness at the first intensity peak was not chosen since manufacturing diamond slabs with thicknesses lower than 100 µm presents significant technological challenges. The recombiners were made to be as solid and stable as possible while offering a large optic area for diffraction. Therefore, they were manufactured with an optic area of 30 mm × 30 mm, a thickness of 25 mm, and with strain relief cuts 2.5 mm wide, using dislocation-free monocrystalline germanium. All the optic surfaces and their lattice planes are required to be very flat, with residual curvature radius ≥2.5 km, to accept the low-divergence XFEL beams (*i.e.* ≥4 µrad for EuXFEL). The maximum residual curvature radius is calculated by dividing the minimum divergence for EuXFEL (4 µrad) by the maximum footprint of a beamlet on the surface of a recombiner (10 mm) obtained for the chosen combinations of splitter (diamond, maximum 3 mm × 3 mm optic area, 100 main face, 113 diffraction planes) and recombiner (germanium, 220 main face and diffraction planes, with 10° asymmetry). The roughness and flatness requirements are standard for crystal optics, with roughness (RMS) ≤1 nm on the scale 10 × 10 µm and flatness ≤1 µm over the entire surface.

The quality of the crystals was analyzed by the high-resolution monochromatic X-ray diffraction rocking-curve imaging technique at the ESRF beamline BM05 (Appendix *D*[App appd]). The diamond splitters performed well during rocking-curve imaging, with good crystalline quality through the surface and the bulk. Germanium recombiners appear to have a rougher surface, even if the quality is uniform and consistent over the whole sample. This rougher surface can be attributed to the brittle structure and reduced hardness of germanium and the less-developed finishing technologies compared with silicon or diamond. While the finishing techniques for germanium surfaces used in this work reach RMS ≤ 1 nm (Zápražný *et al.*, 2015 ▸), diamond surfaces can reach RMS ≤ 0.3 nm (Ovartchaiyapong *et al.*, 2012 ▸) and silicon surfaces can reach RMS ≤ 0.2 nm (Riveros *et al.*, 2019 ▸).

## 5. Mechatronics

Precise six-axis piezo positioners were developed for the multi-projection systems with SmarAct GmbH (Appendix C1). Indeed, the low acceptance of some of the crystal optics calls for very precise and stable crystal alignment. The In-Line geometry has a relatively large tolerance, proportional to the chromaticity of the beam. Indeed, if the angle between the direct beam and a splitter changes, the splitter still diffracts X-rays, just with a slightly different energy within the spectrum of the pink beam. However, the acceptance of the Bragg angle of the recombiners is particularly small (Section 3.2.3). For this reason, the stability and repeatability of the 6-axis positioners were tested via an interferometric system (Appendix C2). The stability measures resulted in an angle drift within 3 µrad over a holding period of 64 h (Fig. 15). From the simulations, these conditions are sufficiently stable conditions to align crystalline optics (Section 3.2.2). The repeatability of the six-axis positioners was also tested, and found to be within 230 nrad (Appendix C2), thus highly reproducible.

## 6. Experimental demonstration

### 6.1. In-Parallel geometry – demonstration

**Figure 7:**
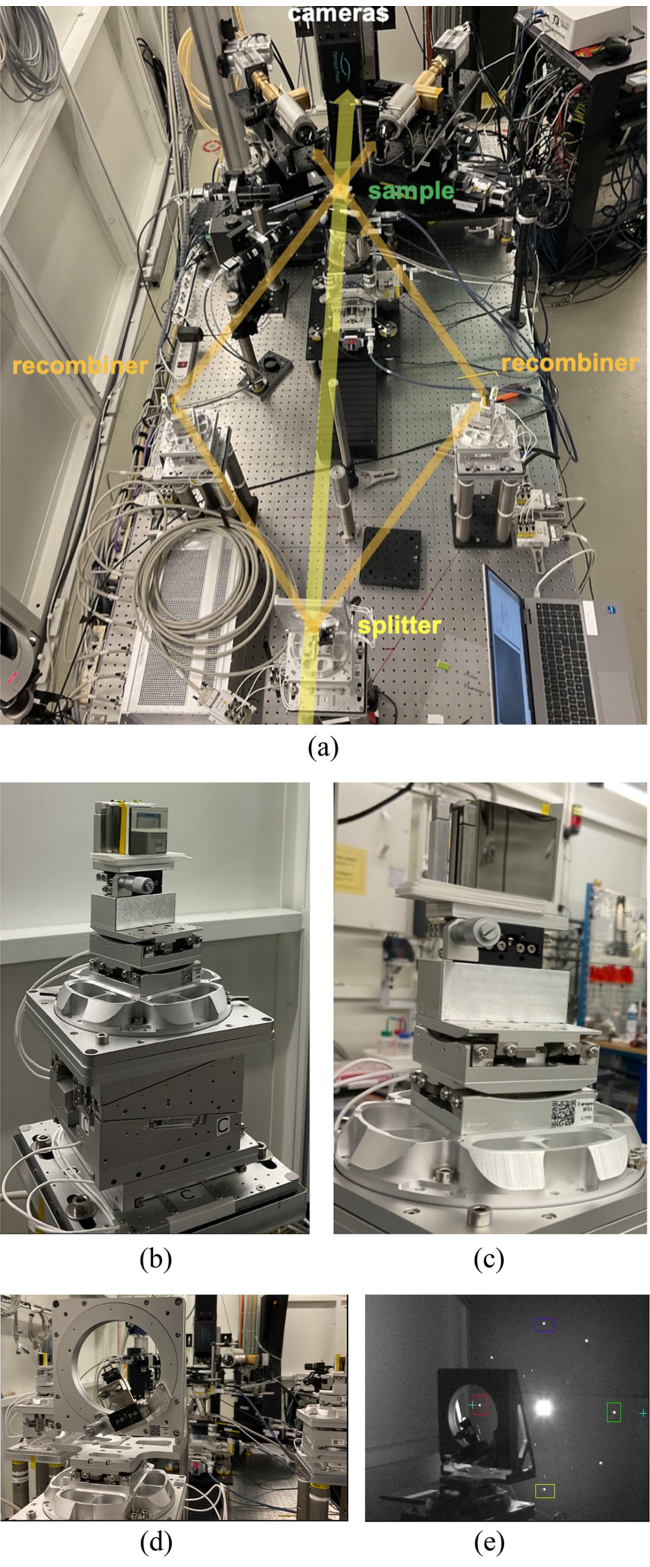
Picture of the In-Parallel setup during the experiment at the PSI TOMCAT beamline. (*a*) Overview of the entire setup. (*b*, *c*) Horizontal recombiners on their six-axis piezo positioners. (*d*) Diamond splitter mounted on its positioner. (*e*) Diamond splitter in diffraction position with the X-ray beam shining through. The direct beam and the eight diffracted beamlets from the (113) plane are visible on a scintillator screen placed behind the splitter.

**Figure 8:**
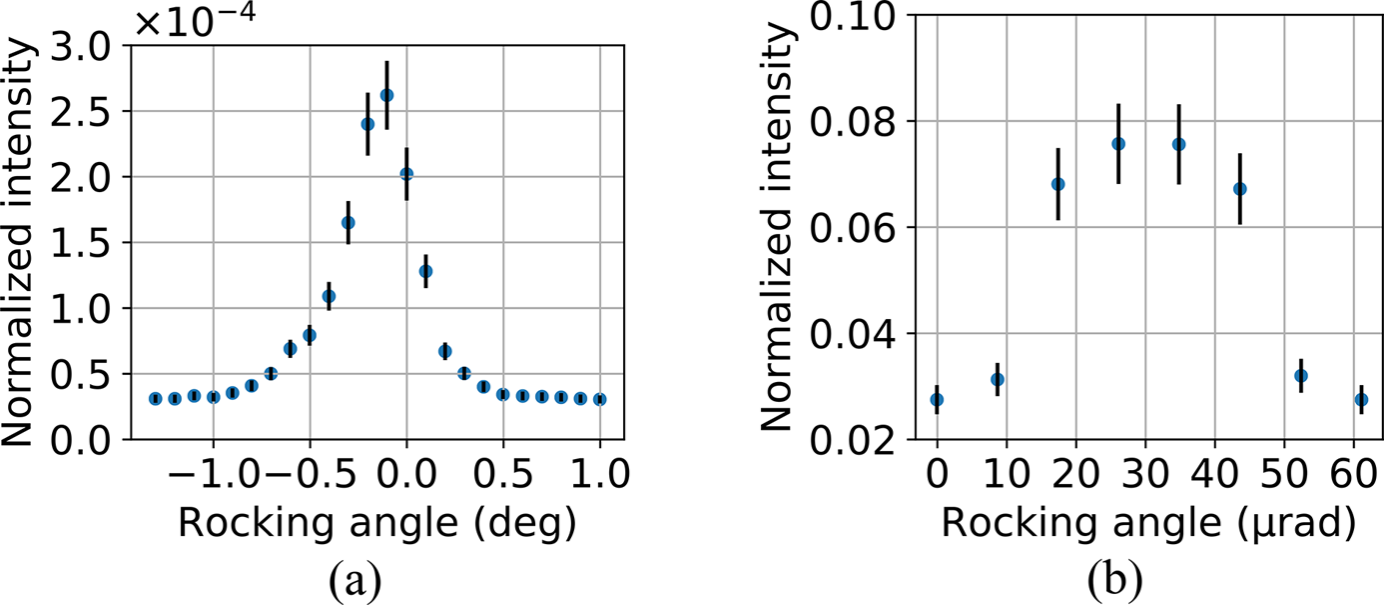
Rocking curves of (*a*) the diamond splitter via one of the beamlets diffracted by the (113) Laue planes with 17.5° asymmetry, (*b*) the germanium recombiner via diffraction on the (660) Bragg planes with 10° asymmetry. (*a*) is normalized by the intensity of the direct beam before the splitter, while (*b*) is normalized by the intensity of the beamlet emerging from the splitter. The error bars are calculated by combining the noise of the detector and the quantum noise for the direct beam, the beamlet after the splitter, or after the recombiner.

The In-Parallel system was tested at the Swiss Light Source synchrotron at the TOMCAT beamline via a pink beam, with a chromaticity of 10^−2^ and an energy of 19.1 keV, to meet the diamond (113) splitter requirements. The splitter was placed to intercept the direct beam and aligned to the position for simultaneous diffraction of eight beamlets, as shown in Fig. 7 ▸. The two horizontal positioners were aligned to intercept the beamlets exiting the splitter. By using the (660) diffraction planes of the recombiners, the beamlets were redirected to a common point intercepting the direct beam. In this case, the beam flux provided by the bending-magnet beamline was too low to enable the acquisition of good images of a sample. However, we recorded the rocking curves of all the crystals by using a diode. Rocking curves are shown in Fig. 8 ▸ for the (660) germanium recombiners and for the (113) diamond splitter. The diffraction efficiency of the splitter is about 70% of what we expected from the simulations, with 2.6 × 10^−4^ measured versus 3.7 × 10^−4^ simulated. This discrepancy is probably due to a larger chromaticity and divergence of the direct beam compared with the simulation. For the recombiners, the diffraction efficiency is 0.075 measured versus 0.21 simulated, so about 36% of the expected value. This larger discrepancy is probably due to the imperfect surface of the recombiners (Fig. 18), which appears rugged when observed at a microscopic level (Appendix *D*[App appd]). Combining the diffraction efficiencies of splitter and recombiners, the resulting measured intensity of each beamlet is 2.0 × 10^−5^ of the beamline flux versus the simulated 7.8 × 10^−5^.

### 6.2. In-Line geometry – demonstration

**Figure 9:**
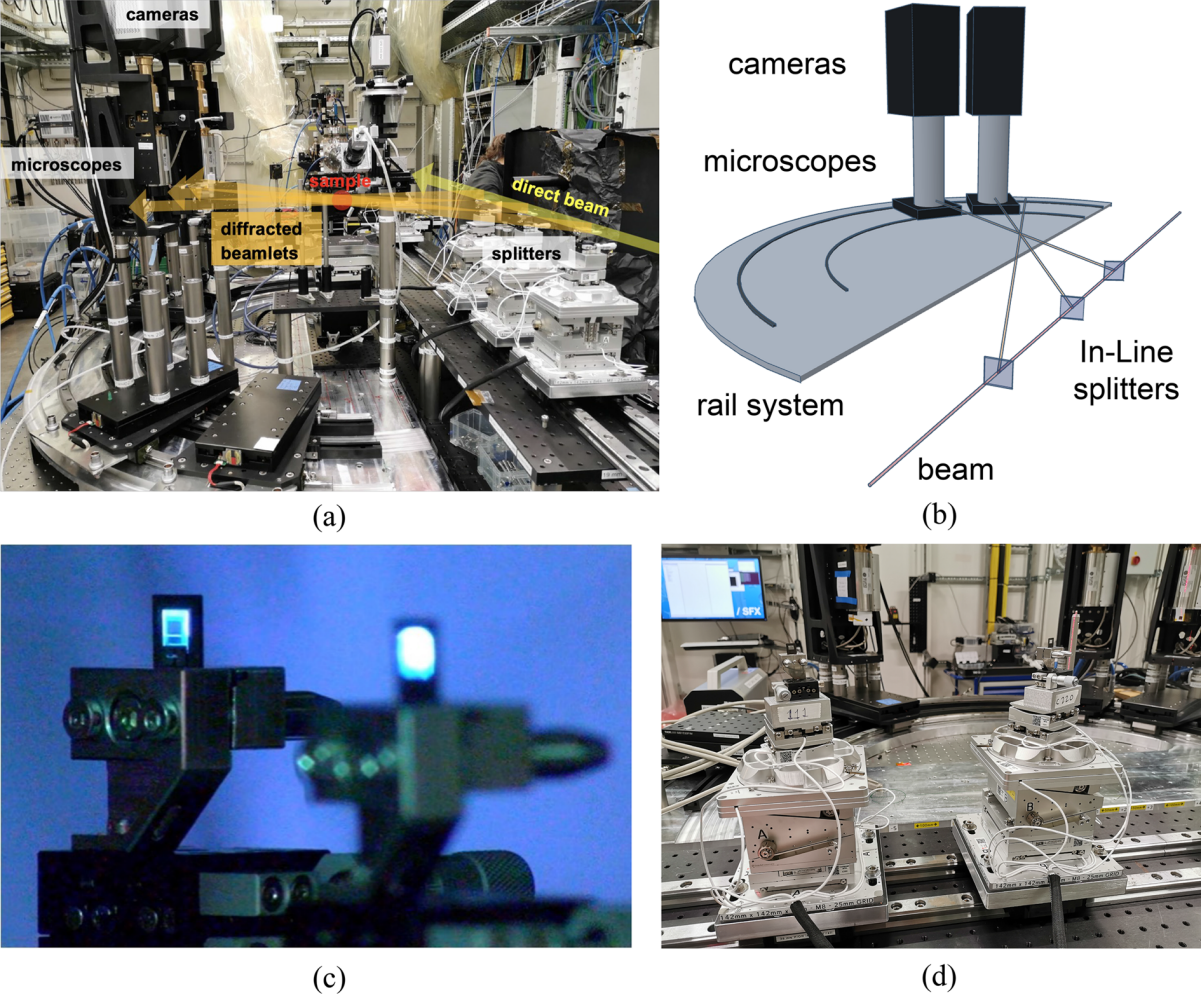
(*a*) Picture of the In-Line setup realized at EuXFEL during the experiment. (*b*) Drawing of the mechatronics for the MHz cameras. (*c*) Two diamond splitters glowing under the illumination of the EuXFEL beam. (*d*) Part of the setup during construction and preliminary testing, with two of the six-axis crystal positioners in the foreground and the camera positioners in the background.

The In-Line geometry (Fig. 9 ▸) was tested at the SPB/SFX instrument of the EuXFEL (Mancuso *et al.*, 2013 ▸; Mancuso *et al.*, 2019 ▸). The photon energy is set to 10 keV, with 10 trains per second, each train containing a number of X-ray pulses chosen by the operators between 1 and 300, each pulse delivering on average 3.3 mJ. The spectrum chromaticity is about 20 eV and the divergence is below 4 µrad. The beam size is clipped to 2.4 mm × 2.4 mm to remove less uniform parts of the beam. The SASE beam instabilities result in a series of artifacts in the images that must be corrected by image processing (Nieuwenhove *et al.*, 2015 ▸; Birnsteinova *et al.*, 2023 ▸).

The In-Line system is fairly tolerant under a pink beam since slight variations in the crystal orientation would just result in slight variations in the diffracted energy while maintaining the diffraction condition. We first used 110 µm- and 130 µm-thick diamond splitters via the two most intense Bragg diffraction peaks, (111) and (220), oriented in symmetric Laue geometry to maximize the field of view of the splitters. A Laue symmetric (111) silicon splitter 15 µm thick and 30 mm (H) × 50 mm (V) in size realized by INFN (Mazzolari *et al.*, 2014 ▸; Germogli *et al.*, 2015 ▸) was also tested to explore the behavior of a silicon beam-splitter on the intense beam of EuXFEL. From the data of the splitters and the X-ray source, it is possible to calculate the expected flux for each diffracted beamlet by using simulations based on DuMond diagrams as in Section 3.2.2. The expected flux delivered for each pulse is 7.5 µJ mm^−2^ for Si111, 2.8 µJ mm^−2^ for C111 and 0.72 µJ mm^−2^ for C220 splitters.

A six-axis Physik Instrumente hexapod was used for positioning a test sample, a metal needle with a thin thread. The sample center was positioned at 300 mm from the direct beam, the minimum distance to avoid collisions between the mechanics and the motors involved. The locations of the splitters are adjusted to diffract the X-ray beamlet to the center of the sample as calculated by equation (1), with zero being the position closest to the sample and positive in the direction of the source. Therefore, the splitters were positioned at 181 mm for C220, 428 mm for C111 and 713 mm for Si111. The direct beam detector is composed of an Andor Zyla 5.5 sCMOS camera coupled with a 5× M Plan Apo infinity corrected Mitutoyo objective looking at a YAG 50 µm-thick scintillator via a 45° mirror.

**Figure 10:**
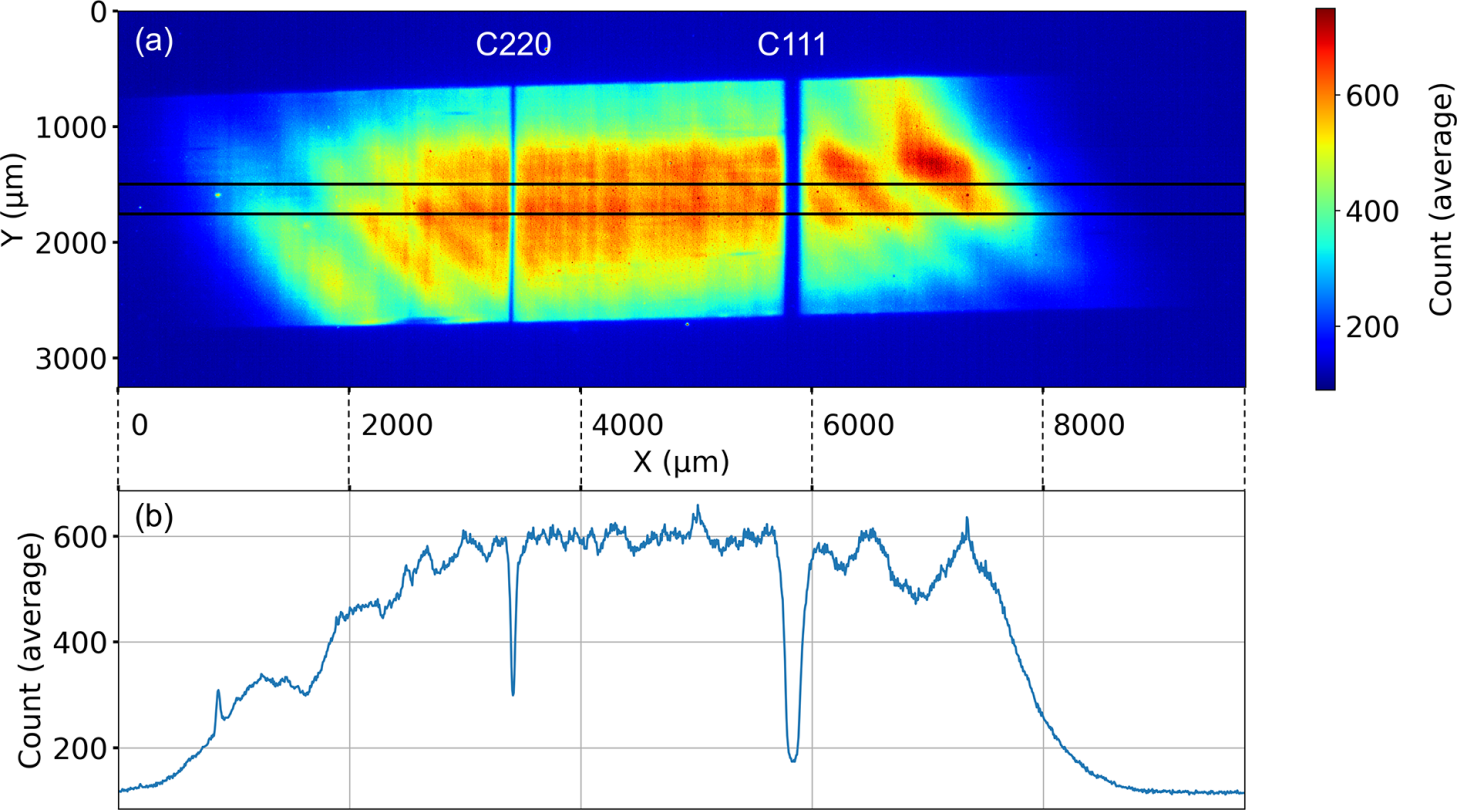
(*a*) Image of the beam spectrum projected by the C333 spectrometer crystal. The image is an average of 15 frames. The parts of the spectrum removed by the C111 and C220 splitters are clearly visible as empty bands. The shape of the beam and non-uniformities are also visible. The part of the image considered for calculating the section in graphic (*b*) is visualized as a black rectangle. (*b*) Section of the spectrometer image over the *X* direction, taken on the center of the image and averaged over 40 pixels in the *Y* direction (260 µm on the detector, as pixel size is 6.5 µm).

The splitters are oriented to the Bragg angle and aligned to the maximum in the diffracted intensity via a spectrometer setup (Boesenberg *et al.*, 2017 ▸; Kaganer *et al.*, 2021 ▸; Petrov *et al.*, 2023 ▸). The spectrometer visualizes the energy spectrum of the transmitted beam, showing the spectrum of the direct beam and those parts of this spectrum that were removed by the splitters and transferred to the diffracted beamlets. Looking at these dips in the spectrum, we can align the splitters to diffract the most intense parts of the spectrum, while simultaneously preventing the splitters superposing, so that each splitter diffracts a different part of the spectrum. Examples of these spectra are given in the work of Boesenberg *et al.* (2017 ▸), Petrov *et al.* (2023 ▸) and in Fig. 10 ▸. In our case, the spectrometer setup is positioned before the direct beam camera and it is composed of a bent diamond (333) crystal diffracting in Bragg geometry part of the transmitted beam onto an X-ray detector, composed of an Andor Zyla 5.5 sCMOS camera coupled with a 50 µm-thick YAG scintillator. The bent crystal offers a different Bragg diffraction angle to every photon energy, so different photon energies are diffracted onto different areas of the camera. Therefore, the image is a direct visualization of the beam spectrum.

Each diffracted beamlet passes through the sample and is intercepted by a camera. The mechatronics of the camera imaging and positioning system were developed by SUNA Precision GmbH (Fig. 9 ▸). The main structure consists of a semi-circular rail with the sample position at its center. The cameras move on the rails, so providing a rough alignment between each camera and a beamlet. The fine alignment between each camera and a beamlet is provided by four motors on the camera base. The imaging plane (scintillator position) of each camera is located 500 mm from the sample. A detailed description of the optical system and the hardware integration such as the fast Shimadzu HPV-X2 and Zyla 5.5 cameras is given by Vagovič *et al.* (2019 ▸).

The image acquisition by the MHz cameras must be synchronized with the train of X-ray pulses. For this purpose, a MicroTCA (MTCA.4 System, MTCA-6P-PH20x) or a set of Stanford Research DG645 delay generators can be used. The camera frames cannot be perfectly synchronized with the X-ray pulses because the camera’s recording speed is specified with a resolution of 10 ns. Our experiment is performed at 1.128 MHz XFEL pulse frequency, so pulses are equally spaced by 886 ns. We, therefore, set the camera speed to 890 ns to approximate the pulse spacing. The mismatch of 4 ns, multiplied by the 128 images in the camera buffer, results in a maximum mismatch of 512 ns inside the train or ± 256 ns. The YAG:Ce scintillator emission reduces from 100% to 10% after about 275 ns following X-ray illumination (Olbinado *et al.*, 2017 ▸). Therefore, we set the camera acquisition window to 590 ns, to prevent capturing two different X-ray pulses in the same camera frame, while keeping the acquisition window as large as possible for capturing a large fraction of each X-ray pulse even at the fringes of the train, when the time mismatch is at its maximum.

**Figure 11:**
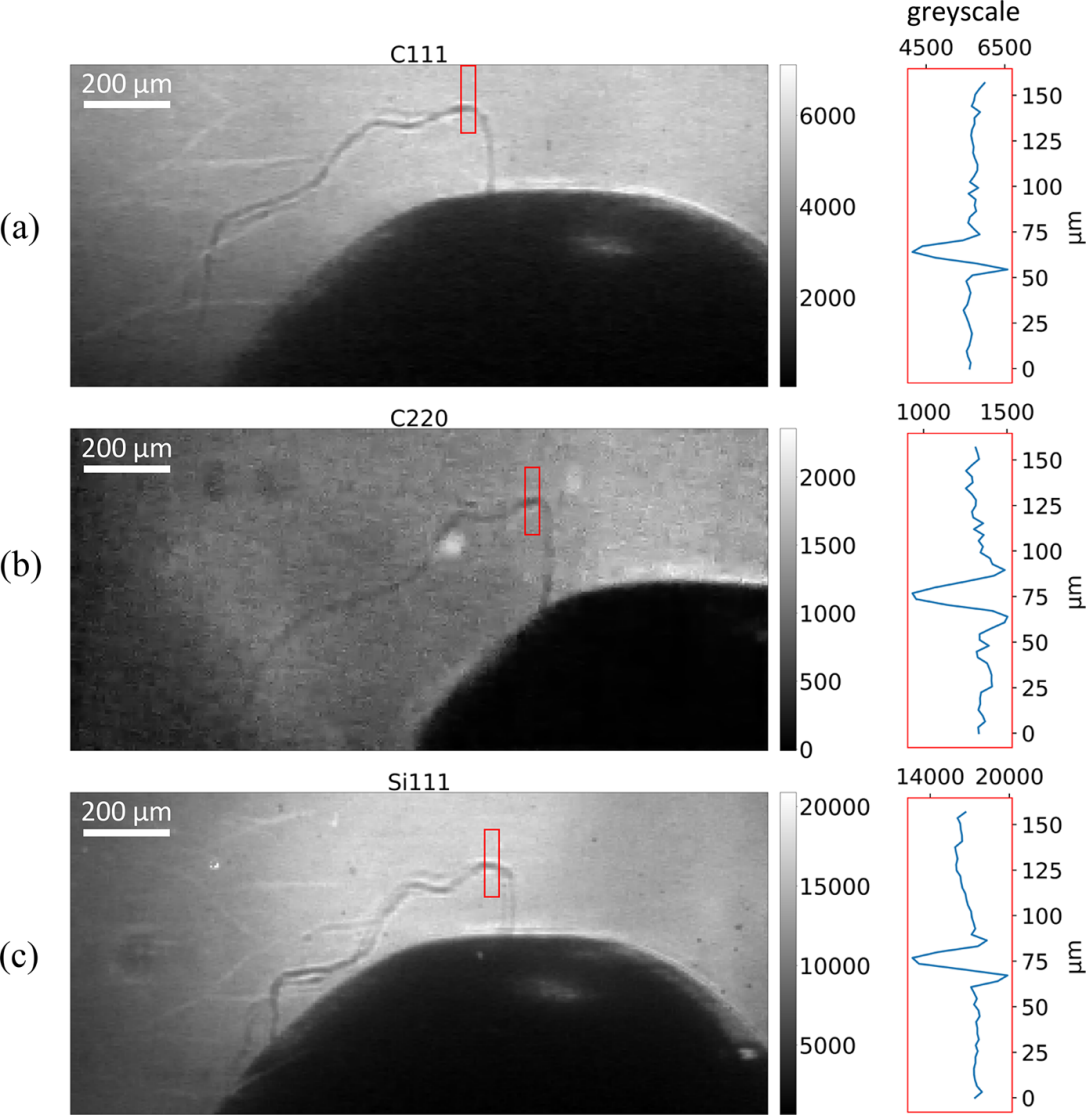
Images of the three projections captured by the 3 MHz cameras with a single pulse, an acquisition time of 590 ns, a repetition rate of 1.128 MHz and 10× magnification. The sample is a metal tip with a plastic fiber thread glued on top. In the red box, a section of the image of the fiber is shown with a height of 160 µm, averaged over the width of 32 µm. The projections are from Laue symmetric splitters diffracting via the lattice planes: (*a*) diamond (111), (*b*) diamond (220), (*c*) silicon (111). We calculate the contrast-to-noise ratio for the detail of the fiber inside the red box, resulting in CNR = 14.1 (*a*), 10.1 (*b*), 30.9 (*c*).

**Figure 15:**
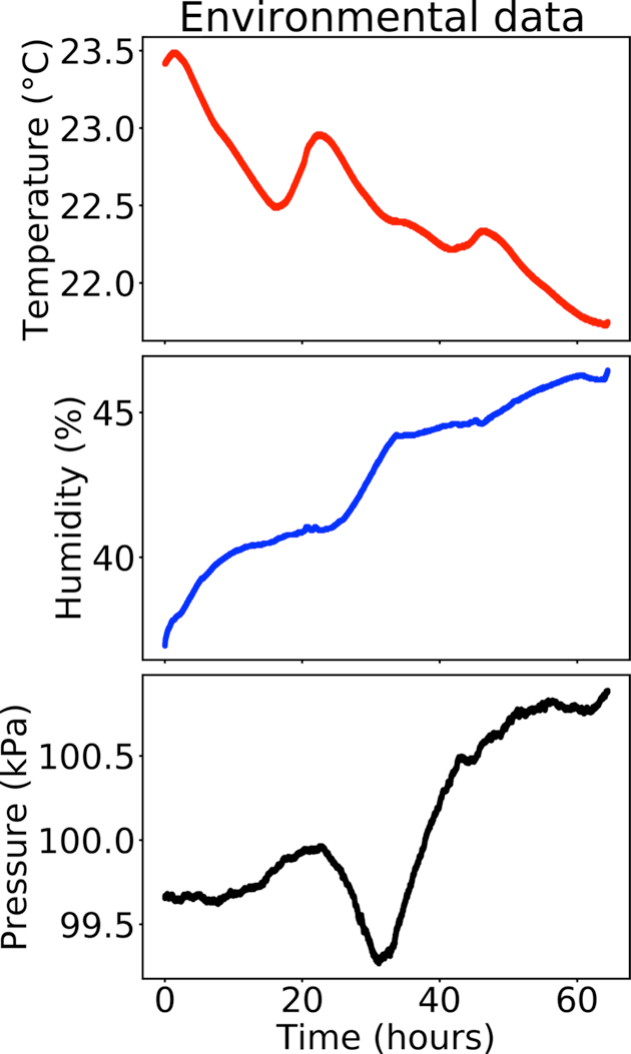
Stability tests on the six-axis piezo positioners over 64 h. Drift of the rotary angle (Bragg angle) at the top, bottom and difference of the two, the latter representing the real drift of the rotary angle when the stability of the structure under the six-axis positioner is eliminated.

**Figure 18:**
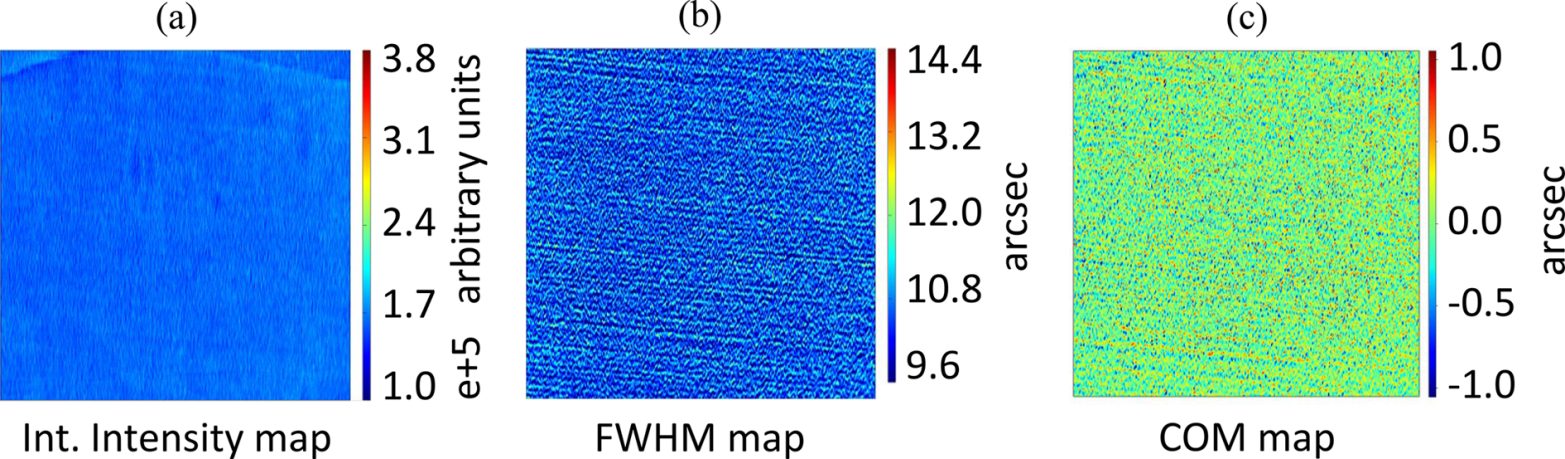
Monochromatic high-resolution X-ray topography at BM05 ESRF beamline for the In-Parallel geometry germanium recombiners. The analysis was conducted via Bragg diffraction on the recombiners’ (440) lattice planes. The images represent an area of the crystal surface 1.3 mm wide horizontally and 5 mm long vertically due to the elongated footprint in the direction of diffraction. The photon energy is 20 keV, the field of view 1.3 mm × 1.3 mm and the pixel size 0.65 µm. (*a*) Integrated intensity map of the surface, *i.e.* map of the intensity diffracted by each point on the surface for a specific Bragg angle of the recombiner. (*b*) FWHM map of the surface, *i.e.* map of the diffraction passband for each point on the surface. (*c*) COM map, *i.e.* map of the relative position of the center of the rocking curve of each point on the surface.

Snapshots from the recorded videos are shown in Fig. 11 ▸ as stereographic images of the sample, full videos are provided in the supporting information. The angles between the beamlets are 23.8° for the C220 and C111 beamlets, and 12.2° for the C111 and Si111 beamlets. The C111 beamlet produces images of good quality, reaching contrast-to-noise ratio (CNR) = 14.1 for the detail of the fiber highlighted in Fig. 11 ▸(*a*). The C220 beamlet is 4.2 times less intense than the C111 beamlet, so its images have a lower, yet acceptable CNR = 10.1 for the same detail in Fig. 11 ▸(*b*). The Si111 beamlet is 3.1 times more intense than C111, resulting in the highest contrast-to-noise ratio, with CNR = 30.9 for the same detail in Fig. 11 ▸(*c*). However, the images from the Si111 beamlet present aberrations in the form of duplicated images, *i.e.* in some of the frames the object appears to be duplicated. This aberration is caused by the large energy passband of Si111 combined with the SASE spectrum, which is composed of a series of sharp peaks (Kujala *et al.*, 2020 ▸) presenting spatial chirping. Even if the FWHM of the angular divergence of the XFEL pulse is 4 µrad, some of the spectral components may exceed this figure and be distributed in space. When two peaks fall inside the Si111 passband, two beamlets emerge at slightly different angles. As a result, the image appears duplicated. Si111 has the widest passband between the splitters, so it has the highest probability of diffracting two peaks.

## 7. Conclusions and outlook

In this paper, we developed crystal optics for fast multi-projection X-ray microscopy and we demonstrated that, via this instrumentation, it is possible to attain multi-projection X-ray imaging up to a frame rate of 1.128 MHz. The presented designs work best at a monochromatic or pink beamline, such as an XFEL beamline with a SASE source. This is due to the narrow passband of the crystal optics efficiently using a beam with a narrow spectrum. We demonstrated the technology enabling multi-projection imaging so that beamlines may offer rotation-free 4D X-ray imaging to their users.

With this new instrument, beamlines around the world may be able to perform 4D imaging on fast or fragile opaque samples that have never been observed before. Our XMPI approach focuses on a small field of view and high temporal resolution, which is complementary to other XMPI systems (Voegeli *et al.*, 2020 ▸) that are more suitable for large beams and tend to a large field of view with slower temporal resolution. Future research for developing the multi-projection technology may focus on stable, thin membrane-like beam-splitters composed of heavier materials to increase the efficiency and luminosity of each projection. Further improvement may also come from aligning the diffraction plane of the system in the vertical plane since horizontal polarization is common in synchrotron or XFEL beams, resulting in small amounts of radiation being diffracted horizontally at Bragg angles near 45°.

## Supplementary Material

Video, corresponding to Figure 10, C111. DOI: 10.1107/S1600577524008488/gy5062sup1.avi

Video, corresponding to Figure 10, C220. DOI: 10.1107/S1600577524008488/gy5062sup2.avi

Video, corresponding to Figure 10, Si111. DOI: 10.1107/S1600577524008488/gy5062sup3.avi

## Figures and Tables

**Figure 12:**
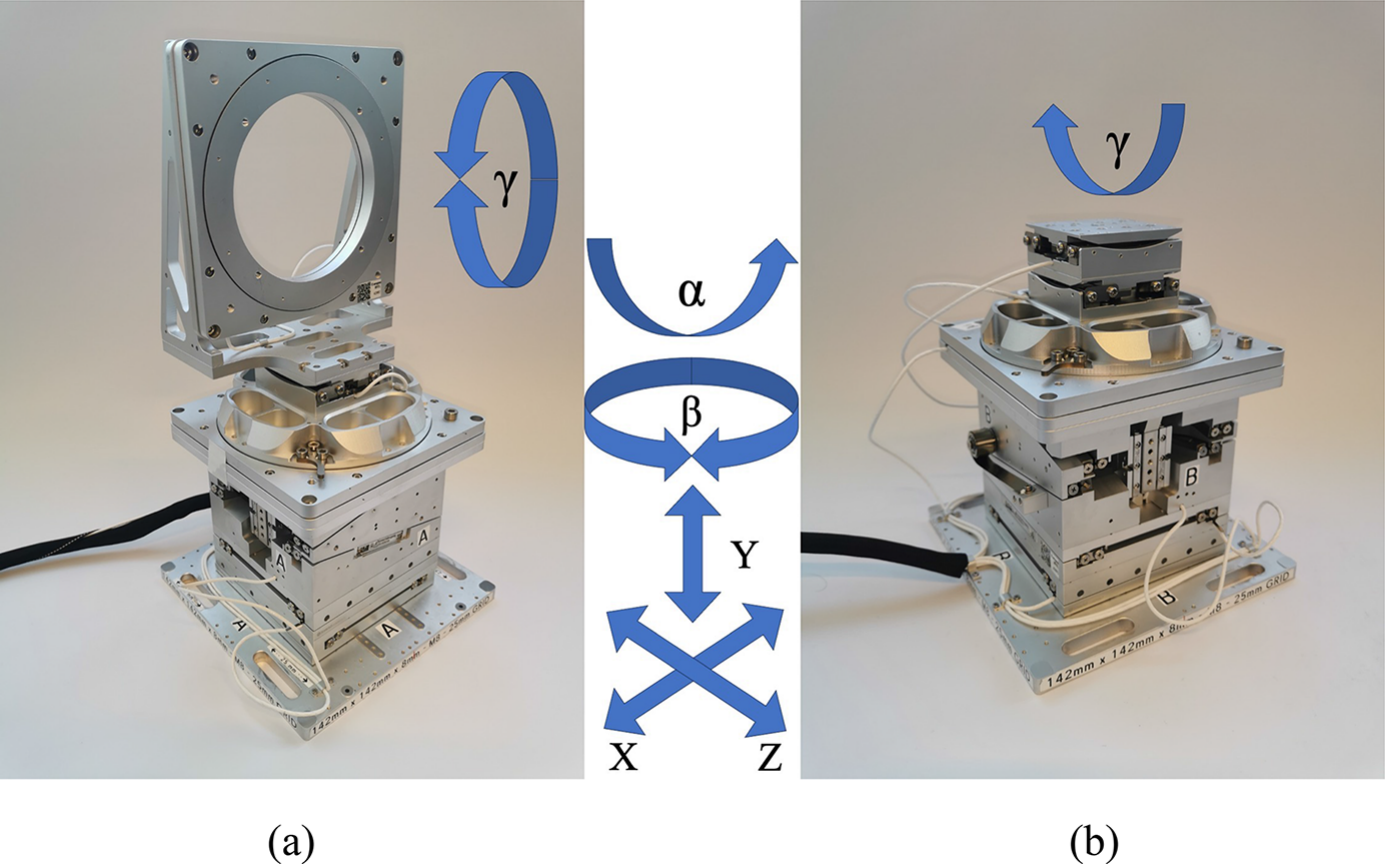
Picture of two models of the six-axis piezo positioners used for aligning the crystals. For both models shown in (*a*) and (*b*), the first five motors are identical. From the bottom up these are: two horizontal linear motors (*X*, *Z*), a vertical motor (*Y*), a rotation around the vertical axis (β), and a tilt (α). The top motor (γ) is a rotary motor in (*a*) and a tilt in (*b*). (*a*) is used as positioner A and C, while (*b*) is used as positioner B (Appendices C1.1 and C1.2).

**Figure 13:**
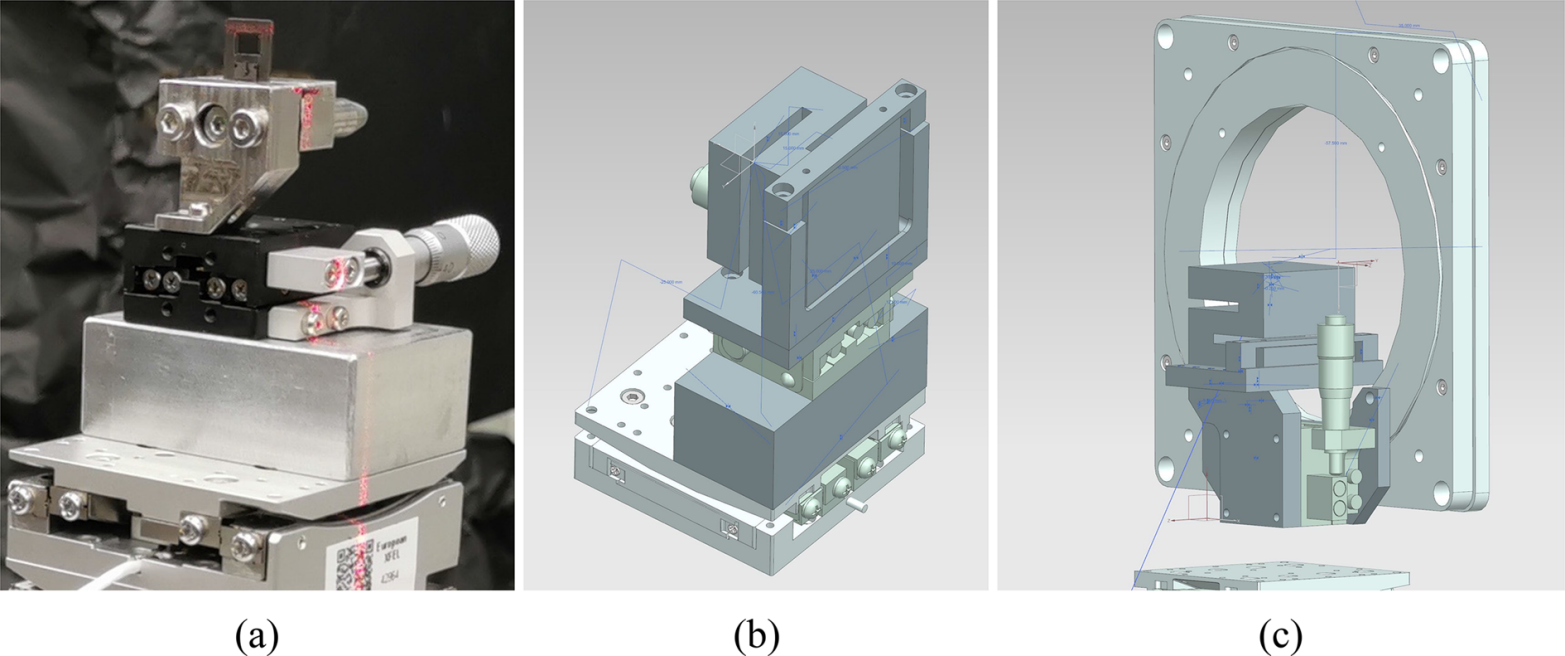
(*a*) Picture of a beam-splitter being pre-aligned to the beam with a laser and the manual stage on top of the six-axis piezo positioners. (*b*) CAD drawing of the horizontal recombiner holder. (*c*) CAD drawing of the vertical recombiner holder.

**Figure 14:**
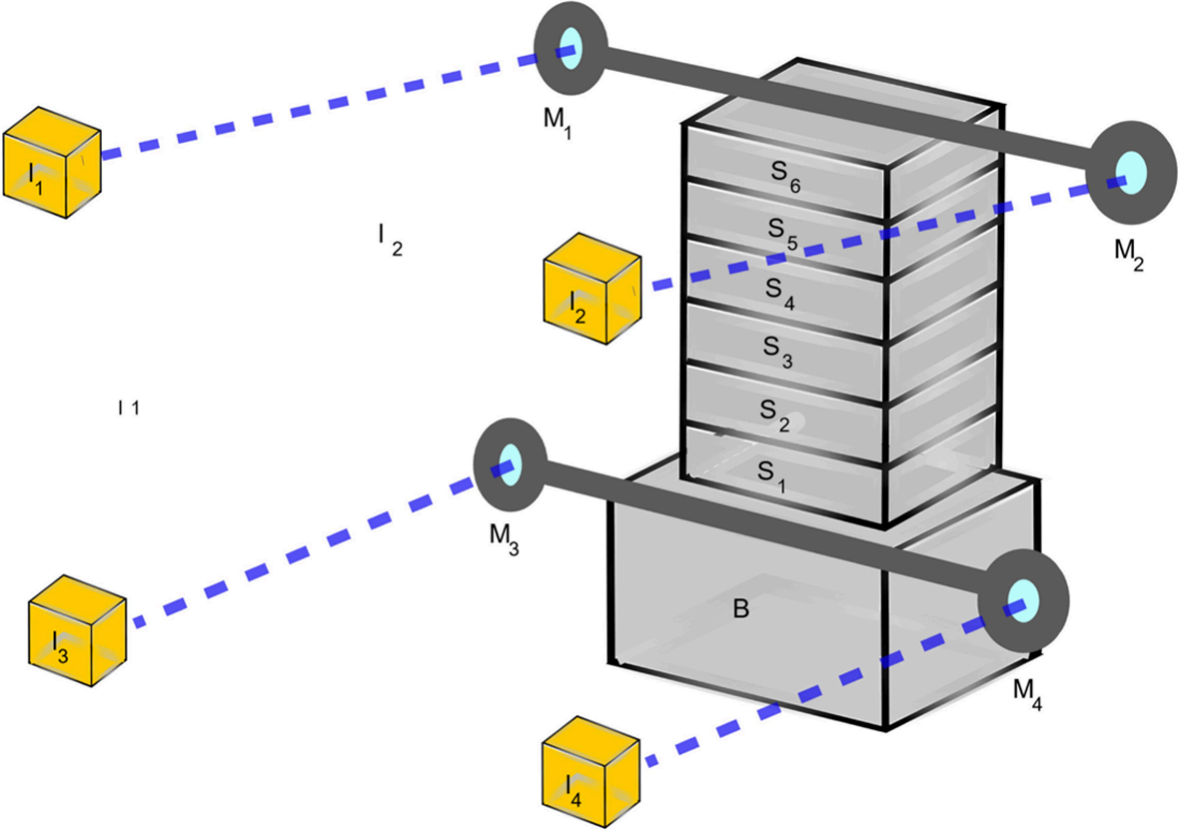
Sketch of the interferometric setup used for the stability and repeatability measures on the six-axis piezo positioners, with *I*_*n*_ the interferometric units, *M*_*n*_ the mirrors, *S*_*n*_ the motors and *B* the base. Two bars with mirrors at the end were affixed to the top and bottom of the positioner. The difference in position between the two mirrors at the ends of one bar gives the rotary angle, which controls the Bragg angle.

**Figure 16:**
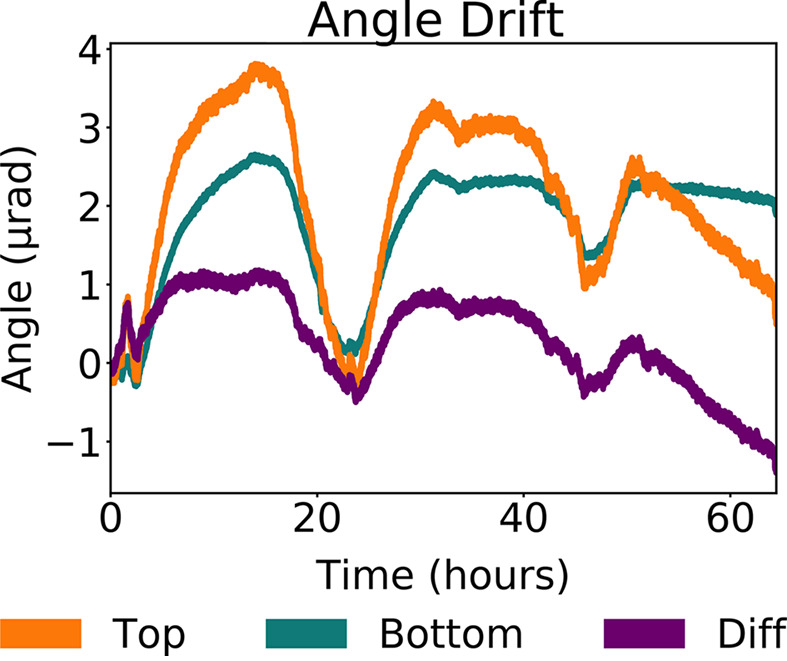
Environmental data during the stability tests on the six-axis piezo positioners, spanning 64 h.

**Figure 17:**
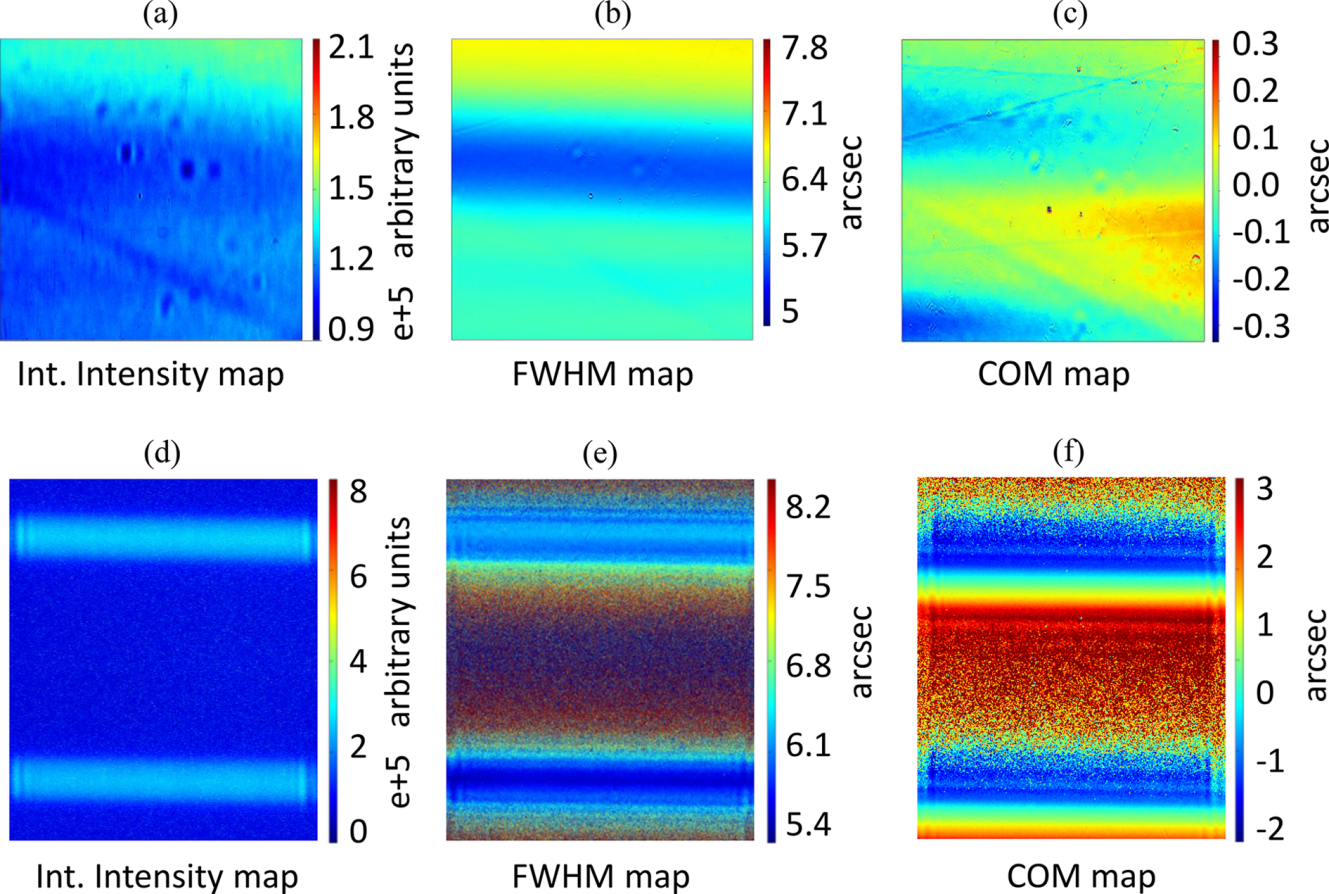
Monochromatic high-resolution X-ray topography at BM05 ESRF beamline on a diamond beam-splitter. The analysis was conducted via Laue diffraction on its (220) lattice planes, both on the (220) main face and the (220) orthogonal to the main face. The photon energy is 20 keV, the field of view 1.3 mm × 1.3 mm and the pixel size 0.65 µm. (*a*, *b*, *c*) are images of the crystal surface, image size 1.3 mm × 1.3 mm. (*a*) Integrated intensity map of the surface, *i.e.* map of the total intensity diffracted by the rocking curve of each point on the surface. (*b*) FWHM map of the surface, *i.e.* map of the diffraction passband for each point on the surface. (*c*) Center of mass (COM) map, *i.e.* map of the relative position of the center of the rocking curve of each point on the surface. (*d*, *e*, *f*) are magnified images of two crystal sections, the distance between the sections being 500 µm. They are section topography maps through the splitter, (*d*) integrated intensity map, (*e*) FWHM map, (*f*) COM map.

**Table 1:** Design simulations for the thickness and size of diamond, silicon and germanium splitters in the In-Line geometry as from the dynamical theory of X-ray diffraction The size of clear diamond splitters is limited to 3 mm × 3 mm by technology, while silicon splitters can be larger. The diffraction geometries chosen are symmetric Bragg or Laue diffraction. The lattice planes with Miller indices from (111) to (440) are selected. The simulated photon energies are between 8 and 15 keV.

	Diffraction plane						
	111	220	311	400	422	333	440
Diamond							
Laue thickness (µm)	100–120	50–100	20 or 70	15 or 85	12 or 100	12 or 100	30
Bragg thickness (µm)	25	40	50	60	70	70	80
							
Silicon							
Laue width (mm)	3.1	3.3	3.4	3.7	4.2	4.5	5.1
Laue thickness (µm)	10	8	10–12	10	10	10	10
Bragg width (mm)	23	14	12	10	8	8	7
Bragg thickness (µm)	9–10	3–5	3–5	4	5	8	8
							
Germanium							
Laue width (mm)	3.2	3.4	3.6	4.1	5.3	6.1	9.3
Laue thickness (µm)	4	4	5	4	4.5	4.5	4.5
Bragg width (mm)	18.3	11.2	9.6	8.0	6.5	6.1	5.6
Bragg thickness (µm)	0.6	1	2	2	2.5	2.5	2.5

**Table 2:** Selection of beam-splitters for the In-Parallel geometry The properties of families of diffraction planes with cylindrical symmetry are studied. Multiplicity represents the number of lattice planes in that particular family and symmetry conditions, therefore the number of beamlets that a family can originate. Some combination of main surface and diffraction plane can appear at multiple asymmetry angles, *e.g.* the combination with main surface (111) and diffraction plane family {135} appears at 3 different asymmetry angles.

Diffraction plane	Multiplicity (number of planes)	Asymmetry Bragg angle (°)	Energy for Si splitter (keV)	Energy for C splitter (keV)
Main surface (100)				
{111}	4	35.26	3.42	5.21
{113}†	8	17.55	12.56	19.10
{133}	4	13.26	21.70	33.00
{224}	8	24.09	16.30	24.80
{244}	4	19.47	20.55	31.26
{115}	4	11.10	30.82	46.89
{135}	4	9.73	39.95	60.78
{155}	4	8.05	58.21	88.56

Main surface (110)				
{220}	4	30.00	6.46	9.82
{113}	6	25.24	8.88	13.52

Main surface (111)				
{113}	3	10.02	21.75	33.08
{135}	6	43.09	9.89	15.04
	6	17.02	23.07	35.09
	6	5.6	69.20	105.27

† The {113} family of planes was selected for the In-Parallel setup.

**Table 3:** Angle of view between two opposite beamlets (θ_V_, in degrees) for the In-Parallel geometry, considering the 311 diamond or silicon 8-beam-splitters and silicon or germanium recombiners The recombiner diffraction planes are looped over the higher orders of the 100, 110 and 111 planes. 110-oriented recombiners were selected (highlighted in bold) because they present large angles of view with changing splitter, with some angles near 90°.

	Si recombiners		Ge recombiners	
Recombiner diffraction plane	Si 311 splitter	C 311 splitter	Si 311 splitter	C 311 splitter
{400}	15.10°	–	11.52°	–
{800}	116.43°	44.04°	106.90°	39.06°
{12 0 0}	–	113.07°	–	103.80°
**{440}**	**53.59°**	8.83°	**48.12°**	5.54°
**{660}**	**131.17°**	**51.68°**	**120.88°**	**46.30°**
**{880}**	–	**99.96°**	253.17°	**91.66°**
{10 10 0}	–	160.54°	–	146.66°
{333}	42.56°	2.16°	37.66°	–
{444}	85.95°	27.63°	78.59°	23.47°
{555}	137.53°	54.47°	126.15°	48.95°
{777}	–	115.52°	–	106.06°

**Table 4:** Results of the repeatability tests of the six-axis positioners, performed by moving the rotary motor in steps over a range of positions, with step size and range size changing All the repeatability data are for 1σ, 10 target positions, and 25 repetitions for each target position.

	Travel range		
Criterion	1.74 µrad	17.4 µrad	174 µrad
Maximum reversal error (nrad)	30.39	107.32	230.97
Unidirectional repeatability (nrad)	49.47	50.11	178.32
Bidirectional repeatability (nrad)	50.06	70.38	200.5

## Appendix A Dynamical theory of X-ray diffraction

We calculate the integrated diffracted intensity  for symmetric Laue or Bragg diffraction by following the dynamical theory of X-ray diffraction (Authier, 2001 ▸).

### A1. Laue diffraction

The integrated diffraction intensity in Laue geometry  is calculated (Authier, 2001 ▸, p. 98, equation 4.40) as

where *C*_p_ is the polarization factor, χ_*h*_ is the dielectric susceptibility of the diffraction plane, γ = γ_*h*_/γ_*o*_ the asymmetry factor (Authier, 2001 ▸, p. 84, equation 4.24), θ_B_ the Bragg angle, *t* the thickness of the splitter, Λ_L_ the extinction length in Laue geometry for the specific diffraction plane and asymmetry factor, *J*_0_(*z*) the zeroth-order Bessel function,  the transmission of the diffracted beam, with μ the linear absorption coefficient of the material; γ_*o*_ and γ_*h*_ are, respectively, the direction cosines of the incident and diffracted beam relative to the inner normal to the crystal surface,

where β is the angle between the normal to the crystal surface and the trace of the lattice planes (Authier, 2001 ▸, p. 82, equation 4.11). β = 0 for symmetric Laue diffraction and β = −π/2 for symmetric Bragg diffraction. The asymmetry angle can be defined as β in Laue geometry and α = β + π/2 in Bragg geometry. Since β = 0 in symmetric Laue diffraction, γ_*o*_ = γ_*h*_, γ = 1 and the absorption factor is reduced to .

The  function versus the thickness of the Laue splitters follows *Pendellösung* oscillations, *i.e.* the oscillation in intensity between the transmitted and diffracted beam due to the symmetric nature of Laue diffraction. The maximum  is reached when the thickness is comparable with the extinction length, *i.e.* the length over which virtually all the beam is diffracted.

### A2. Bragg diffraction

In the Bragg case, there is no exact formula to calculate  over a wide range of thicknesses. However, since Bragg diffraction does not present strong *Pendellösung* oscillations, the integrated diffracted intensity converges rapidly to an average (Authier, 2001 ▸, p. 103, equation 4.47*a*),

where *t* is the thickness of the crystal Λ_B_ is the extinction length for the Bragg geometry, δ_os_ is the Darwin width, *i.e.* half the angular acceptance of the lattice plane for Bragg diffraction,

where *C*_p_ is the polarization factor, *r*_e_ is the electron radius, λ is the wavelength, *V* the volume of the unit cell, γ = γ_*h*_/γ_*o*_, *F*_*c*_ the structure factor for the particular diffraction plane with Miller indices *hkl* or . In the case of symmetric Bragg geometry, β = π/2, γ_*h*_ = −γ_*o*_ and |γ| = 1. The integrated diffracted intensity in Bragg geometry is higher than in Laue geometry because of the reduced thickness that the diffracted beam must traverse.

From the Darwin width and Bragg’s law 2*d* sin |θ_B_| = *n*λ = *hc*/*E*, it is possible to calculate the energy acceptance of the lattice plane for diffraction as the range of energies diffracted within its angular acceptance,

where *d* is the interplanar distance, λ is the wavelength of the diffracted radiation, *h* is the Planck constant, *c* is the speed of light, *E*_max_ and *E*_min_ the maximum and minimum energy within the Darwin width.

## Appendix B Magnification

For the recombiners, the asymmetry angle is defined as the angle α between the lattice planes and the physical surface of a crystal. A grazing-incidence angle can be used for enlarging the acceptance of the crystal [equation (13)] (Authier, 2001 ▸) while enlarging the physical size of the diffracted beamlet over the diffraction direction by a magnification factor *M*,

where θ_in_ and θ_out_ are the incident and outgoing angles between the beamlet and the recombiner surface, θ_B_ is the Bragg angle, Δθ_oc_ and Δθ_hc_ are, respectively, correction terms for the incoming and outgoing beam obtained by the dynamical theory of diffraction (Authier, 2001 ▸), and α is the asymmetry angle. In our setup, enlarging the beamlet’s physical size can be beneficial since the beamlet was already shrunk due to the asymmetry of the splitter, by a factor of 0.818 for both the C113 and Si113 splitters. The total magnification of the beamlet is obtained by multiplying the magnifications produced by the splitter and the recombiner. Therefore, we can select a recombiner asymmetry that increases the acceptance while making the shape of the beamlet more symmetric, or similar to the shape of the field of view of the camera. Cameras often have a larger horizontal field of view. As an example, the MHz camera Shimadzu HPV-X2 used in this study has a field of view of 400 horizontal (H) × 250 vertical (V) pixels, an aspect ratio H/V of 1.6. Therefore, we can adjust the magnification to approximate this value to have beamlets that fit the field of view of the camera. In our specific case, this optimization leads to selecting a grazing asymmetry of 10° because the magnification factor for the selected 110 planes (Table 3 ▸) re-balances the shrinking caused by the splitter and produces beamlets with an aspect ratio similar to the field of view of the camera. For a germanium recombiner with 10° asymmetry, at 19.1 keV the magnification is 3.11, 1.93, 1.52 for (440), (660) and (880) diffraction planes, respectively, resulting in a total magnification of the image of 2.55, 1.58, 1.24. At 12.55 keV the magnification is 1.90 and 1.38 for (440) and (660) diffraction planes, respectively, resulting in a total magnification of the image of 1.56 and 1.13.

## Appendix C Mechatronics

### c1. Motor assembly

The low acceptance of some of the crystal optics, in particular the recombiners of the In-Parallel geometry, calls for very precise and stable mechanics for beam alignment and keeping the alignment stable over the duration of an experiment. For this purpose, we developed together with SmarAct GmbH precise six-axis positioners composed of six stacked motors. The order of arrangement is important since the positioners must be able to align the crystal lattice planes with the rotation angle controlling the Bragg angle. This is particularly important for the In-Parallel splitter since it must meet multiple diffraction conditions, so two orthogonal rotation axes must be functionally independent. Consider a reference system with a horizontal *x* axis, a vertical *y* axis and a *z* axis aligned in the beam direction. α, β and γ are the rotation angles around these axes, respectively. All the positioners require the same base platform composed of five motors, from bottom to top: two linear horizontal axes (*XZ*), a vertical linear axis (*Y*), a rotation around the vertical axis (β) and a tilt. The final motor of the positioner varies depending on the specific optics it will be used with, such as a recombiner, a splitter In-Parallel mode, β In-Line mode.

#### c1.1. In-Parallel geometry

The positioners can be divided into three types according to the function of the mounted optics.

A – Laue splitter positioner [Fig. 12 ▸(*a*)]. The β rotation aligns the Bragg angle of the beamlets in the horizontal plane, the α tilt aligns the Bragg angle of the vertical beamlets, and the γ rotation aligns their angle around the beam. The adjustments in α and β are critical since they control the Bragg angle; therefore, any misalignment in these angles could cause the splitter to go out of diffraction.

B – horizontal recombiner positioner [Fig. 12 ▸(*b*)]. The Bragg angle is regulated by the β rotation stage. In addition to the rotation around angle β, two additional tilts around α and γ are required to adjust the diffracted beamlets.

C – vertical recombiner positioner [Fig. 12 ▸(*a*)]. These positioners are identical to type A but rotated by 90° around the vertical axis. In order to align the beamlet in the vertical direction, the positioners of type C affect a tilt along γ. In this case, the Bragg angle is regulated by the top α rotation stage, also used for aligning the recombiner to different diffraction orders, *e.g.* (220), (440), (660).

#### c1.2. In-Line geometry

In this geometry, all optical components diffract a single beamlet over the same diffraction plane. However, two different positioner types are needed in case different splitters are used. In both types, the Bragg angle is regulated by the β rotation stage.

A – skew planes positioner [Fig. 12 ▸(*a*)]. This positioner is identical to type ‘A – splitter positioner’. It is used to align skewed planes, *i.e.* planes non-parallel to any of the sides of the splitter. The large γ rotation aligns the skewed plane with the horizontal diffraction plane. As for the previous positioner type, if the diffraction plane is vertical, it is necessary to flip the entire assembly composed of the top three rotary motors by 90°.

B – standard In-Line positioner [Fig. 12 ▸(*b*)]. This type of positioner is identical to type ‘B – horizontal recombiner positioner’. The two top tilts around α and γ fine-tune the alignment of the diffraction plane in the horizontal plane. In our experiments, we used a horizontal diffraction plane. However, these positioners can also be utilized for a vertical diffraction plane by rotating the top three rotary motors by 90°. This can be achieved either by using a right-angle bracket or by employing a type C positioner.

#### c1.3. Clamping

In addition to the piezo motor structure described above, custom holders were designed to clamp the crystals and align their diffracting planes in the center of rotation of the positioners with micrometric precision (Fig. 13 ▸). For the splitters, the crystal’s center is placed in the center of the rotation, while for the recombiners, it is the center of the main face. To ensure that the crystals (Fig. 5 ▸) are clamped without experiencing stress in their optic area, stress relief cuts were incorporated (Samoylova *et al.*, 2019 ▸; Kaganer *et al.*, 2021 ▸). In this design, the clamping occurs on the side opposite the optic surface, while deep cuts separate the clamped portion from the optic surface. The stress resulting from clamping is distributed in the material within the stress relief cuts, which is the thinnest and longest part of the crystal. Consequently, any deformation occurs in this specific region. This deformation causes a net rotation of the optic part, but it does not introduce any curvature or other deformations to the optic part itself. The rotary motors of the six-axis positioner can easily compensate for this net rotation.

### c2. Stability and repeatability tests

Analysis of stability and repeatability was performed by interferometric measurement with 20 nrad resolution. The interferometric system was composed of two ‘Picoscale’ interferometers coupled with an aluminium bar with mirrors at the extremities, so the angular displacement is calculated knowing the length of the bar and the movement of the mirrors. One interferometric system was mounted on top of a piezo six-axis positioner (Fig. 14 ▸), while another was mounted on its base. Temperature, humidity and air pressure were monitored in the room during the measurement. The stability of the system was measured over intervals spanning a maximum of 64 h. During this measure, environmental data such as temperature, pressure and humidity were measured (Fig. 15 ▸). The stability of the six-axis positioner resulted in less than 3 µrad displacement over 64 h (Fig. 16 ▸), which gives sufficiently stable conditions to align crystalline optics from the simulations (Section 3.2.2). The repeatability of the system was also tested (Table 4 ▸) by the reversal error, unidirectional and bidirectional repeatability. When aiming to reach a target position *X*_T_, the reversal error is the difference between the actual position *X*_P_ reached and the target position *X*_T_, when approached from opposite directions. In our case, the maximum reversal error for each travel range 1.74, 17.4, 174 µrad was extracted from an ensemble of motions to 10 target positions and 25 repetitions for each target position. The travel ranges were chosen to be realistic travel ranges for rocking-curve alignment and optimization. Directional repeatability is a measure of the positioning system’s ability to sequentially reach the same position *X*_P_ when aiming to reach the target position *X*_T_. It can be unidirectional when always approaching the target position from the same direction, or bidirectional when approaching the target position from either direction. It is measured by collecting an ensemble of positions *X*_P*i*_ reached by the system and computing their standard deviation, *i.e.* the sigma of their histogram. The results show that the system is highly reproducible even for the largest range of 174 µrad. Bidirectional and unidirectional repeatability are within 200 nm, while the maximum reversal error is within 230 nrad.

## Appendix D X-ray diffraction imaging

The quality of the crystals was analyzed by high-resolution monochromatic X-ray topography at the ESRF beamline BM05 (Tran Thi *et al.*, 2017 ▸; Ziegler *et al.*, 2004 ▸). This includes rocking-curve imaging and section topography to investigate both the surface and the bulk of the crystals. The beamline was set to a 20 keV monochromatic beam after a double-diffraction (111) silicon monochromator. Different diffraction planes were analyzed to test the crystal quality for dislocations that can appear over particular directions (Fig. 17 ▸). These images were taken with a high-resolution detector with a field of view of 1.3 mm × 1.3 mm and a pixel size of 0.65 µm, stitching the entire optic surface of the crystals.

The diamond splitters (shown in Fig. 17 ▸) performed well during rocking-curve imaging, with good crystalline quality and FWHM 5.5 arcsec. A map of the optic surface also shows some polishing waves, within tolerance. The variation of the center of the rocking curves visible in the center of mass (COM) map is much smaller than the FWHM of the rocking curve.

Section scans were performed by taking three sections from one surface to the opposite surface in order to analyze the bulk of the crystal. During section topography, the beam is limited by slits spaced by a distance of 500 µm from each other. One slit enables the passage of a beam 1.3 mm wide and 10 µm high. This technique produces an image where the defects illuminated by a slit are distributed spatially on a 2D image (in our case in the vertical direction) as a function of their position through the depth of the crystal (Tran Thi *et al.*, 2017 ▸). In our case, the sections are quite uniform and the section scans show no evident defects or inclusions in the bulk.

The topography of the recombiners shows a different picture (Fig. 18 ▸). Germanium recombiners appear to have a rougher surface, even if the quality is uniform and consistent over the whole sample. In this case, section scans are not possible because of the thickness of the sample. This rougher surface can be attributed to the brittle structure and lower hardness of germanium and the less-developed finishing technologies compared with silicon or diamond.
